# Energy Consumption vs. Tensile Strength of Poly[methyl methacrylate] in Material Extrusion 3D Printing: The Impact of Six Control Settings

**DOI:** 10.3390/polym15040845

**Published:** 2023-02-08

**Authors:** Nectarios Vidakis, Markos Petousis, Nikolaos Mountakis, Amalia Moutsopoulou, Emmanuel Karapidakis

**Affiliations:** 1 Department of Mechanical Engineering, Hellenic Mediterranean University, 71410 Heraklion, Greece; 2 Electrical and Computer Engineering Department, Hellenic Mediterranean University, 71410 Heraklion, Greece

**Keywords:** Poly[methyl methacrylate] (PMMA), optimization, material extrusion (MEX), energy consumption, energy efficiency, compressive strength, taguchi analysis, robust design

## Abstract

The energy efficiency of material extrusion additive manufacturing has a significant impact on the economics and environmental footprint of the process. Control parameters that ensure 3D-printed functional products of premium quality and mechanical strength are an established market-driven requirement. To accomplish multiple objectives is challenging, especially for multi-purpose industrial polymers, such as the Poly[methyl methacrylate]. The current paper explores the contribution of six generic control factors (infill density, raster deposition angle, nozzle temperature, print speed, layer thickness, and bed temperature) to the energy performance of Poly[methyl methacrylate] over its mechanical performance. A five-level L25 Taguchi orthogonal array was composed, with five replicas, involving 135 experiments. The 3D printing time and the electrical consumption were documented with the stopwatch approach. The tensile strength, modulus, and toughness were experimentally obtained. The raster deposition angle and the printing speed were the first and second most influential control parameters on tensile strength. Layer thickness and printing speed were the corresponding ones for the energy consumption. Quadratic regression model equations for each response metric over the six control parameters were compiled and validated. Thus, the best compromise between energy efficiency and mechanical strength is achievable, and a tool creates significant value for engineering applications.

## 1. Introduction

For the material extrusion (MEX) 3D printing process, research on the mechanical properties of parts built with polymers, such as Acrylonitrile Butadiene Styrene (ABS) [1,2,3], Polycarbonate (PC) [4,5,6,7], Polylactic Acid (PLA) [8,9,10], Polyamide 12 (PA12) [11,12,13,14,15], Thermoplastic Polyurethane (TPU) [16,17], polyester [18] is extensive. Polymers have been studied in the pure form [3,4,8,11,16,19] and as matrices in composites [9,12,18]. Commonly used approaches to study polymers’ effects on material extrusion manufacturing processes are both theoretical (e.g. numerical calculations) [20,21,22] and experimental methodologies [23,24,25,26]. For the analysis of the results various modeling tools are often used, such as the Taguchi design [3,8,9,15,16,17,19], full factorial Design of Experiments [27,28], Box-Behnken design [13], Analysis of Variances (ANOVA) [11,29,30], and artificial neural networks modeling [12]. For the MEX 3D printing process research on its sustainability is still limited and related to the energy efficiency of the process [31,32].

Poly[methyl methacrylate] (PMMA) is a thermoplastic widely used in energy storage [33,34,35,36,37] and medical applications, focused on cranioplasty [38,39,40,41,42] and dentistry [43,44]. Its antimicrobial properties have also been investigated [44,45,46]. In these areas composites using PMMA as the matrix material have also been developed, aiming to improve the performance of the polymer. Additives, such as silica, carbon, and boron nitride have been employed [47,48,49,50,51,52,53]. The sustainability of PMMA has been studied, focusing on its performance when recycled in 3D printing [54] and elsewhere [55,56,57,58,59]. For the analysis and optimization of PMMA performance, modeling tools have been employed [60,61,62,63]. In the field of application of the PMMA, the 3D printing process can be easily adapted, with its characteristic providing additional merit to the applications. As expected, research has been expanded to this process as well, i.e., energy and dental applications implemented with 3D printing with the PMMA polymer [64,65,66,67,68,69,70,71]. Studies are aiming to investigate the mechanical properties of the 3D printing parts [64,66,68], and their antibacterial performance [67,71]. Apart from these application areas, PMMA has been investigated in Hybrid Additive Manufacturing, in combination with the Friction Stir Welding process [72], and overall research for the PMMA polymer is rather limited in 3D printing so far.

Sustainability is considered a critical aspect affecting climate change [73,74]. There is an increasing concern about the consequences on the environment and the adoption of sustainable practices [75]. The effect of the manufacturing processes on sustainability is investigated [76]. Energy consumption is among the critical parameters affecting the sustainability of the processes [77,78]. Additive Manufacturing (AM) is considered a sustainable process, due to the reduced energy requirements when compared to other manufacturing processes. Other parameters to support the sustainability of the AM process is the reduced waste [79,80], and the use of recycled materials [81,82]. Research on the sustainability of the AM process is extensive, investigating how it is affected by the process parameters [83], environmental factors [84,85,86], etc.

The sustainability aspects of the PMMA polymer in 3D printing have not been investigated so far. Parameters such as the energy consumption during the manufacture of the parts with the MEX 3D printing process are critical for the sustainable production of parts. Research has shown that energy consumption is affected by the 3D printing parameters used [32]. At the same time, 3D printing parameters highly affect the mechanical properties of the 3D printed parts [11,13,19]. This work aims to provide insight into the energy consumption of parts built with the PMMA polymer with the MEX 3D printing process. Tensile test specimens were manufactured, and their mechanical properties were studied. The possibility of optimizing both the energy consumption and the mechanical properties, by selecting a set of 3D printing settings that optimizes both metrics was investigated. A Taguchi L25 orthogonal array was formed, and experimental findings were further processed with ANOVA. Prediction models were compiled as functions of the six 3D printing settings investigated herein. Their accuracy was evaluated with two confirmation runs and it was shown that they are more than sufficient for real-life applications use. Optimizing both the energy consumption during the MEX 3D printing process of the PMMA-made parts and their mechanical properties in tensile tests were not possible. It was found though, that parts with increased tensile strength had moderate energy demands. Overall, this study is aiming to achieve the following research goals:

- Quantify the energy demands when manufacturing parts with the PMMA polymer, with the MEX 3D printing process.
- Identify the effect of six generic, machine-independent 3D printing settings on the energy consumption and the mechanical properties of PMMA parts in MEX 3D printing.
- Analyze and optimize the energy consumption and the mechanical properties of these six 3D printing settings studied, aiming to provide a road map for future use.
- Contribute to the Increase of the sustainability of the MEX 3D printing process and the increase of the performance of the parts build with the process.

## 2. Materials and Methods

### 2.1. Materials

**Figure 1:**
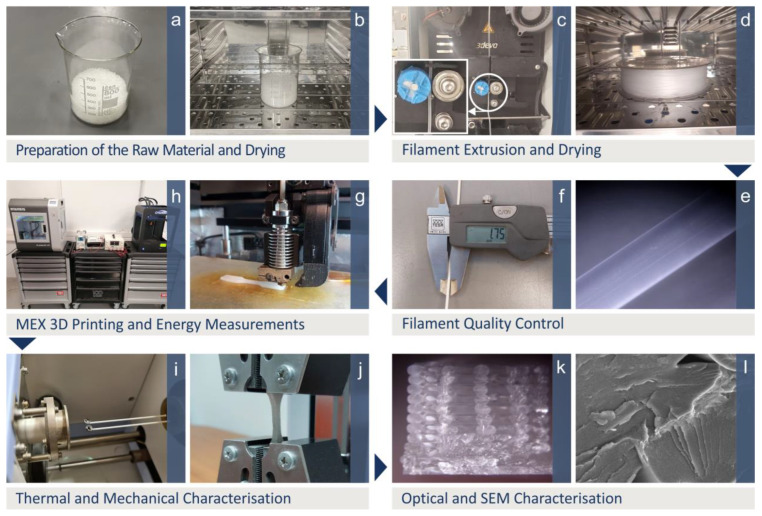
Experimental process. (**a**) PMMA in powder form, (**b**) drying of the PMMA powder, (**c**) PMMA filament extrusion, (**d**) PMMA filament drying, (**e**,**f**) filament quality control, (**g**) samples MEX 3D printing, (**h**) energy measurements, (**i**) thermal-properties determination, (**j**) mechanical testing, and (**k**,**l**) morphological characterization.

PMMA was procured in pellet form (brand name JULIER, Fujian, China, density 1.15–1.19 g/cm^3^, type suitable for extrusion, Figure 1a) and it was dried in a laboratory oven at 40 °C for 4 h before any further use (Figure 1b). It was then extruded into a 1.75 mm filament, compatible with the MEX 3D-printing process (3devo precision, Utrecht, The Netherlands) (Figure 1c).

### 2.2. Filament Examination and Thermal Properties

Before the MEX 3D-printing process, the produced filament underwent quality control for consistency in its diameter and surface quality (Figure 1e,f). Its thermal properties were investigated with thermogravimetric analysis (TGA) (Perkin Elmer Diamond TG/TDA, Waltham, MA, USA, 40–550 °C, 10 °C/min, nitrogen purge gas, Figure 1i) and differential scanning calorimetry (DSC) (Perkin Elmer Diamond DSC, Waltham, MA, USA, 50–300 °C, 10 °C/min, air atmosphere), to verify that the extrusion temperatures (filament and MEX 3D printing) did not compromise the thermal stability of the PMMA polymer.

### 2.3. Specimens Fabrication

**Figure 2:**
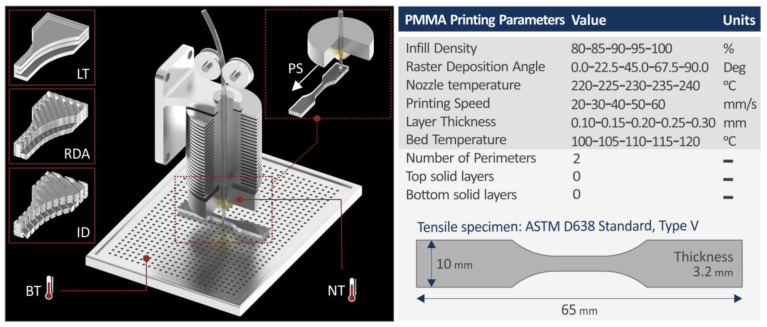
3D-printing process: graphical representation of the 3D-printing settings considered in the work, their values (right side), and the dimensions and the specimens manufactured with the process, following the corresponding standard (ASTM D638-02a).

Prior to the MEX 3D-printing process, filament was further dried (Figure 1d). Tensile test (dogbone) specimens were manufactured with the MEX 3D-printing process with a different set of 3D-printing parameters for each run. Five specimens were manufactured and tested for each run (135 in total). Specimens conformed with the corresponding standard (American Society for Testing and Materials-ASTM D638-02a, type V), and their geometries are shown in Figure 2. An Intamsys, Funmat HT (Intamsys, Shanghai, China) MEX 3D printer was used for the fabrication of the specimens (Figure 1g). The different 3D-printing parameters studied herein are schematically shown in Figure 2, along with their values, which were selected according to the material’s and the 3D printer’s specifications, and the literature [72].

### 2.4. Experimental Process and Samples Characterization

During the specimen manufacturing process, the consumed energy from the 3D printer was recorded with a Rigol DM3058E device (RIGOL Technologies, Shanghai, China). The printing time was also monitored (stopwatch method, [87], Figure 1h). Specimens were then experimentally tested for their properties under tensile loading in accordance with the ASTM D638-02a standard (Imada-MX2 tester, Imada Inc., IL, USA, 10 mm/min elongation speed, Figure 1g). After the mechanical tests, their morphological characteristics were evaluated (side surface quality, fracture mechanism) using an optical stereoscope (Kern OKO 1, 5MP ODC 832 camera, KERN, Balingen, Germany, Figure 1k) and scanning electron microscopy (SEM) (JEOL JSM 6362LV, Jeol Ltd., Peabody, MA, USA, high-vacuum mode, 20 kV, Au sputtered samples, Figure 1l).

### 2.5. Energy Indicators

The energy consumption can be distinguished into three main phases: (i) machine startup, (ii) 3D-printing process, and (iii) machine shutdown, and can be calculated utilizing the equations below [32]:

(1) $$E_{\mathrm{total}}=E_{\mathrm{thermal}}+E_{\mathrm{motion}}+E_{\mathrm{auxiliary}}$$

where:

(2) $$E_{\mathrm{thermal}}=E_{\mathrm{heating}}+E_{\mathrm{cooling}}$$

$E_{\mathrm{motion}}$ is the consumed energy by the motors of the 3D printer and

(3) $$E_{\mathrm{auxiliary}}=E_{\mathrm{startup}}+E_{\mathrm{steadystate}}+E_{\mathrm{shutdown}}$$

of the electronics and the remaining parts of the 3D printer.

The specific printing energy (SPE) index was calculated:

(4) $$\mathrm{SPE}=\frac{\mathrm{EPC}}{w}\;\left[\mathrm{MJ}/g\right]$$

The specific printing power (SPP) index was calculated:

(5) $$\mathrm{SPP}=\frac{\mathrm{EPC}}{\mathrm{PT}\cdot w}\cdot 10^{3}\;\left[\mathrm{kW}/g\right]$$

where energy printing consumption (EPC) represents the energy used by the 3D printer ($E_{\mathrm{total}}$), w the actual weight of each specimen, and PT the actual printing time for each experimental run.

### 2.6. DOE and Regression Analysis

The Taguchi design of experiment was followed in the work [88] and an L25 orthogonal array was formed. Six 3D printing settings were studied for their effect on the energy consumption and the tensile properties of the MEX 3D printed parts, made with the PMMA polymer. The control parameters were selected according to the literature review, while their levels, as mentioned above, were selected according to the material’s and the 3D printer’s specifications and the literature [72]. The control parameters were, Raster Deposition Angle (RDA, deg), Layer Thickness (LT, mm), Infill Density (ID, %), Nozzle Temperature (NT, °C), Bed Temperature (BT, °C), and Printing Speed (PS, mm/min). Each control parameter had five levels and five replicas were tested per run.

The effect of these control parameters on eight response metrics related to the energy consumption during the 3D printing process (MEX 3D printing time—s, manufactured specimens’ weight—g, EPC—MJ, SPE—MJ/g, and SPP—KW/g), and the tensile properties of the PMMA MEX 3D printed specimens (tensile strength—MPa, the tensile modulus of elasticity—MPa and tensile toughness—MJ/m^3^), was investigated. The experimental results were statistically analyzed and then a regression analysis followed for the formation of predictive models for each one of the response indicators, as functions of the six control parameters studied herein. Two additional confirmation runs with five replicas each were performed for the evaluation of the prediction models produced.

## 3. Results

### 3.1. Thermal Properties and Morphological Analysis

**Figure 3:**
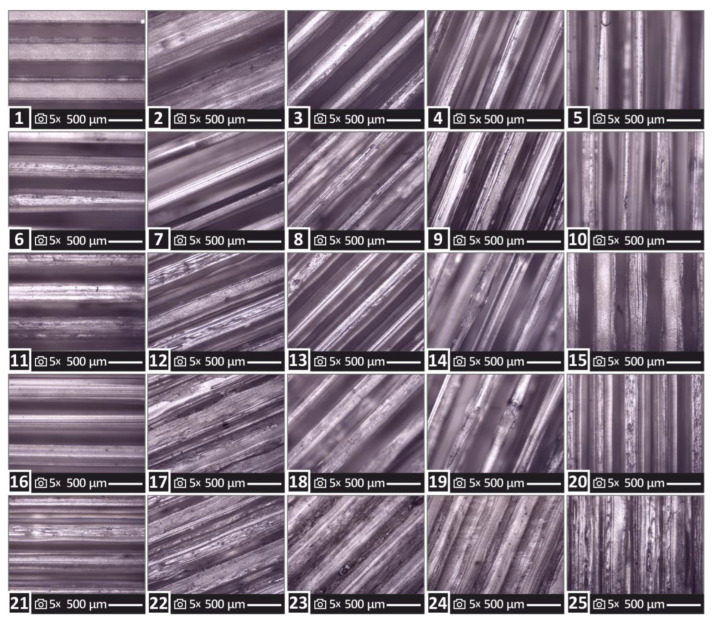
Images from the optical stereoscope of the top surface of one of the five samples for each one of the twenty-five runs studied in the work. The differences in the samples’ 3D-printing structure are visible in the images. The number in the images is the corresponding run from which the image was taken.

One out of the five samples of each run studied herein is presented in Figure 3. The top surface of each specimen is shown and images were acquired using an optical stereoscope. The differences in the 3D-printing structure between the specimens, due to the different 3D-printing parameters used in each run, are evident in the images. In all samples, a defect-free 3D-printing structure is shown, verifying that the 3D-printing parameters used and studied in the work are appropriate for the PMMA material and the 3D printer used.

**Figure 4:**
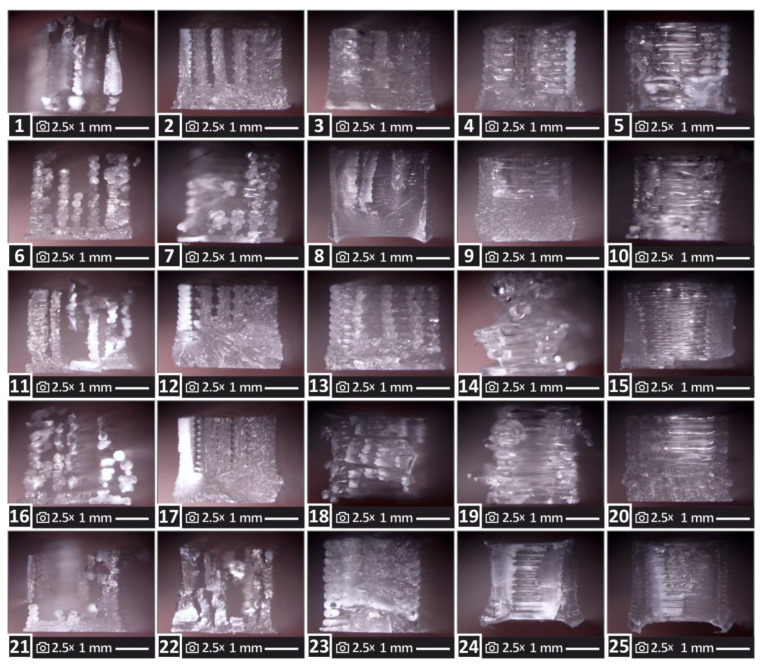
Images from the optical stereoscope of the fracture surface of one of the five samples for each one of the twenty-five runs studied in the work. The number in the images is the corresponding run from which the image was taken.

Figure 4 shows the corresponding images from the fracture surface of the samples (again images were taken with an optical stereoscope). The fracture surfaces significantly differ between the runs, showing the high effect of the 3D printing parameters on the behavior of the 3D printed parts. In some cases (for example run no 3, 17, and others), the fracture surface indicates a brittle failure with minimum deformation shown. In other samples (for example run no 5 and 10) a more ductile fracture behavior is observed, with deformation shown in the 3D printing structure. There are samples with mixed fracture surfaces, such as the samples of run 9 and 12. In several cases, the strands have failed in a brittle manner, but they have also been detached from each other (failure of the fusion between them) and parts of the sample have been abscinded (for example runs 1, 7, 11, and 21). The expected voids and pores in the 3D printing structure [23] are present in the samples, and filament tearing [89] is not visible in the images. In the specimens in which the fusion between the strands seems to have collapsed or partly collapsed (for example in the sample of run 12) the size of the voids is increased and can be attributed to the failure of the specimen during the tensile test. Such abnormalities in the 3D structure affect the mechanical properties of the parts [90].

**Figure 5:**
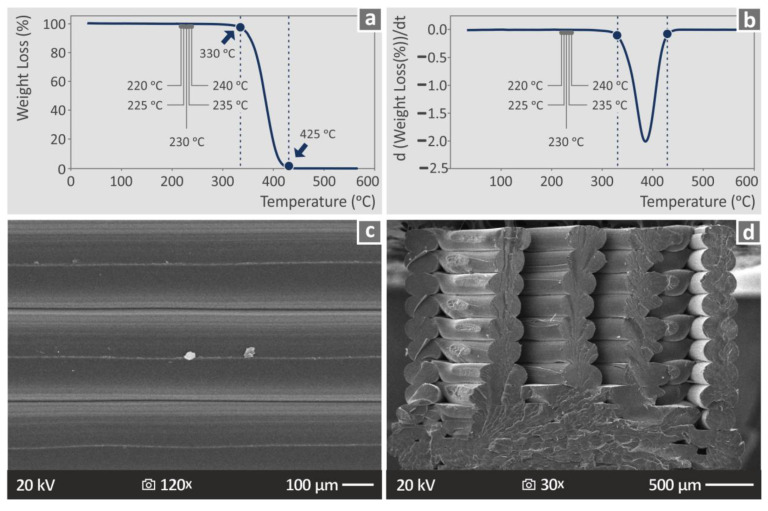
(**a**) TGA weight loss (%) vs. temperature (°C) graph produced during the study of the thermal properties of the PMMA polymer used, (**b**) corresponding d(weight loss)/dt graph produced with DSC, and SEM images from (**c**) the side surface, and (**d**) the fracture surface produced after the completion of the tensile test of a PMMA MEX 3D-printed sample.

The thermal properties of the PMMA polymer, as they were determined during the measurements taken, are shown in Figure 5 (TGA graph Figure 5a, DSC graph Figure 5b). From the derived curves, it is safe to assume that the temperatures applied to the PMMA material during the filament extrusion and the MEX 3D-printing process did not affect its thermal stability or cause any degradation in the material that would affect the produced parts. Higher magnification SEM images from the side surface of the samples (Figure 5c) show a defect-free 3D-printing structure and verified the images taken with the optical stereoscope. SEM images from the fracture surface (Figure 5d), which are of higher magnification than the optical stereoscope, similarly present the fracture surface. The strands failed in a brittle way with no visible deformation. Strands that are piled one above the other maintained their fusion after the failure of the specimen in the tensile test, while the adjacent piles of strands detached, forming internal voids in the specimens. In the specific specimen shown in Figure 5d (run 13), the bottom part is more solid with minimum pores and no clearly visible 3D-printing structure up to about 1/3 of its height. Then, the structure described above is visible.

### 3.2. Taguchi Design and Experimental Results

**Figure 6:**
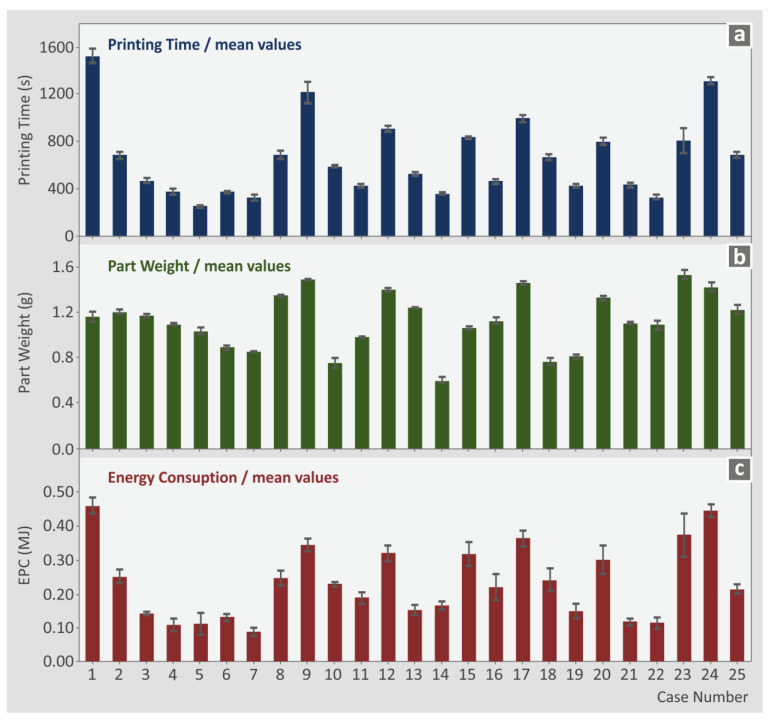
Average values and deviation of the experimental results for each one of the twenty-five runs: (**a**) printing time (s), (**b**) part weight (g), and (**c**) EPC (MJ).

The L25 orthogonal array compiled in the work with the six control parameters with five levels each is presented in Table 1. For each one of the twenty-five experimental runs conducted, the average value and the deviation for each response indicator studied herein is presented in Table 2 and Table 3. Weight, tensile strength (sB), tensile modulus of elasticity (E), and tensile toughness are depicted in Table 2, while Table 3 presents the corresponding values for the printing time, EPC, SPE, and SPP. Figure 6 presents, in a bar chart, the average value and the deviation for the printing time, the part weight, and the EPC for the twenty-five runs. Such a presentation shows in a more vivid way the differences between the cases studied for these indicators, contributing to the comprehensiveness of the variations between the experimental results and the importance of the 3D-printing settings in the performance of the fabricated parts. The experimental results for each specimen are analytically presented in the supplementary material of the work.

### 3.3. Statistical Analysis

**Figure 7:**
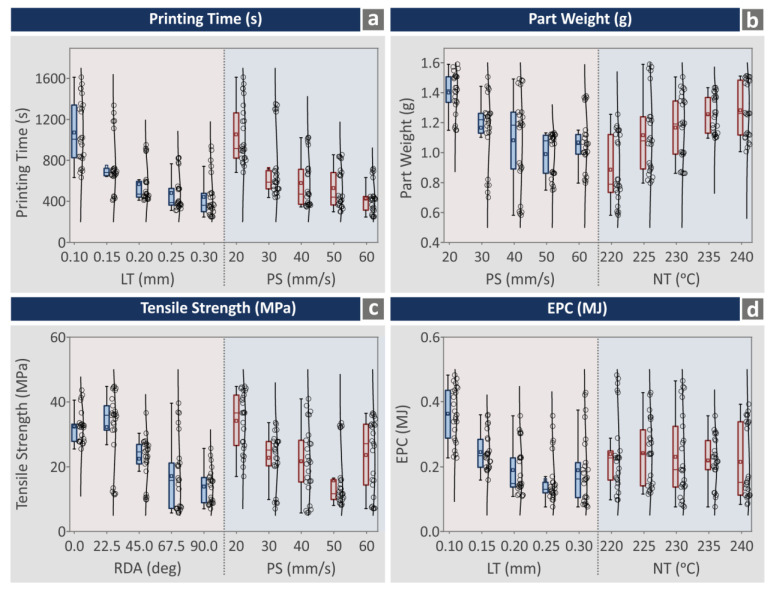
Boxplot graphs (**a**) printing time (s), (**b**) part weight (g), (**c**) tensile strength (MPa), and (**d**) EPC (MJ).

Figure 7 shows boxplot graphs for four of the response metrics studied, i.e., printing time (s), part weight (g), tensile strength (MPa), and EPC (MJ). For each response metric, the boxplots were formed against the two higher ranked control parameters for the specific metric. Scattered experimental-result values for a specific response metric for a given control parameter value indicate that this value has a strong influence on the specific response metric:

- Printing time (s): LT (mm) values result in a scattered pattern, except for the cases of 0.20 mm and 0.25 mm, in which printing-time values are gathered around three different values, which is not a clear compact response. Still, values are rather condensed around these values. For the PS (mm/s) control parameter, printing-time (s) values are scattered in all its levels.
- Part weight (g): both PS (mm/s) and NT (°C) control parameters resulted in a scattered response in the part-weight (g) indicator in all their levels.
- Tensile strength (MPa): both RDA (deg) and PS (mm/s) control parameters caused a scattered response in the tensile-strength (MPa) indicator in all their levels.
- EPC (MJ): both LT (mm) and NT (°C) control parameters caused a scattered response in the EPC (MJ) indicator in all their levels.

The information provided in the boxplots indicates that further analysis is required to have a clearer view of the effect of the 3D-printing settings on the mechanical properties and the energy-consumption metrics.

**Figure 8:**
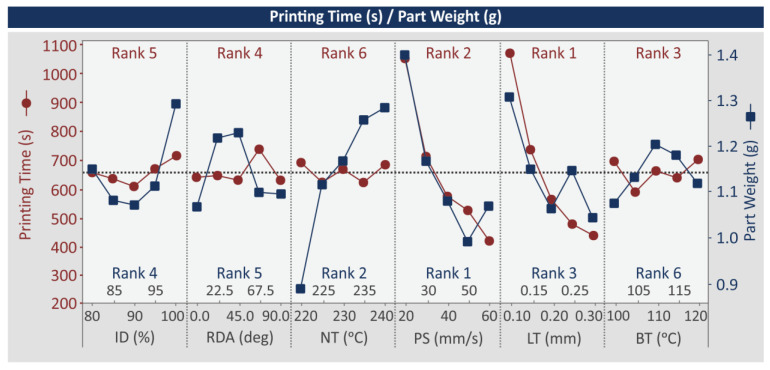
MEP: printing time (s), part weight (g).

Figure 8 shows main effect plots (MEP) for the printing-time (s) and the part-weight (g) metrics. According to Equations (4) and (5), these metrics affect energy efficiency. Printing time (s) is highly affected by the PS (mm/s) and the LT (mm) control parameter only. Higher values in these control parameters lead to reduced printing-time (s) values. LT (mm) is the dominant parameter and PS (mm/s) is ranked no 2. NT (°C) is the least important parameter for the printing time (s). For the part weight (g), PS (mm/s) is the dominant parameter followed by NT (°C) (rank no 2), while BT (°C) is the least significant parameter. Higher PS (mm/s) values decrease the part-weight (g) metric up to the 50 mm/s value. Then, at the 60 mm/s value (the highest studied value for this control parameter), part weight (g) is slightly increased. The increase in NT (°C) increases the part weight (g) metric. None of the other control parameters have a linear response to the part-weight (g) metric, with values initially decreasing and then increasing, as the values increase, or the other way around, showing a significant effect on the specific metric.

**Figure 9:**
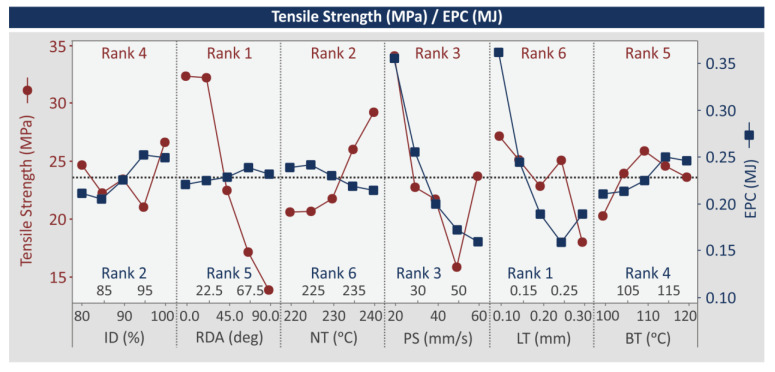
MEP: tensile strength (MPa), EPC (MJ).

Figure 9 presents the MEP for the tensile-strength (MPa) and the EPC (MJ) indicators. This is a crucial plot, since it depicts the set of 3D-printing settings producing the optimum results for strength and energy consumption in the parts built with PMMA, with the MEX 3D-printing process. RDA (deg) was found to be the dominant parameter for the tensile strength (MPa), followed by NT (°C). The increase in RDA (deg) significantly reduces the tensile strength (MPa), while the increase in NT (°C) increases this metric. LT (mm) was the least important control parameter. Its increase reduced the tensile strength (MPa). ID (%) had a zig-zag effect with a decrease at first, then a slight increase, then again a decrease and the highest tensile-strength (MPa) values were found at the highest ID (%) value. The increase in the PS (mm/s) control parameter decreased the tensile strength (MPa) up to 50 mm/s and then it was increased for the 60 mm/s value. Still, the highest tensile-strength (MPa) value was reported for the lowest PS (mm/s) value, 20 mm/s. BT (°C) increased the tensile strength (MPa) up to the median values (110 °C), which resulted in the highest metric values, and then the tensile strength (MPa) started to decrease. For the EPC (MJ) metric, RDA (deg), NT (°C), and BT (°C) have no significant effect on the metric. LT (mm) was the dominant parameter, followed by ID (%), and NT (°C) was the least important parameter affecting the EPC (MJ). The increase in LT (mm) constantly decreased EPC (MJ) up to the 0.25 mm value and then it was slightly increased at the 30 mm LT value. Low ID (%) values result in low EPC (MJ) values. Finally, the increase in PS (mm/s) decreased the EPC (MJ) values. MEP for the remaining response metrics is available in the supplementary material of the work.

**Figure 10:**
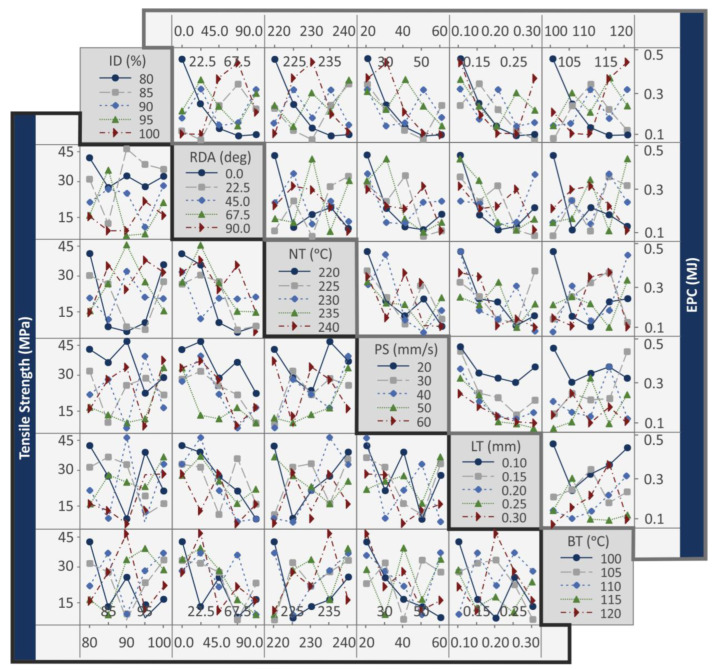
Interaction plots: tensile strength (MPa), EPC (MJ).

MEP provides information about the optimum response values for the metrics evaluated; still, they do not provide any information about the relations between the control parameters in each response indicator. For this purpose, interaction plots were created and are presented in Figure 10 and the supplementary material of the work. Figure 10 shows the interaction plots for the tensile strength (MPa) and the EPC (MJ) metric. In all cases, antagonistic relations between the control parameters are observed. Only in the case of EPC (MJ), is a synergistic relation shown between the PS (mm/s) and the LT (mm) control parameters.

### 3.4. Regression Analysis

The reduced quadratic regression model (RQRM) for each response was calculated:

(6) $$Y_{k}=a_{k}+\overset{n}{\underset{i=1}{\sum }}b_{i,k}x_{i}+\overset{n}{\underset{i=1}{\sum }}c_{i,k}x_{i}^{2}+e_{k}$$

where k represents the quality output (e.g., weight, printing time, tensile strength, tensile modulus of elasticity, tensile toughness, EPC, SPE, SPP); a is the constant value; b is the coefficient of the linear terms; c is the coefficient of the quadratic terms; e is the error; and x_i_ the seven (n = 6) control parameters, i.e., the infill density, raster deposition angle, nozzle temperature, printing speed, layer thickness and bed temperature.

For each response metric, a regression table was formed along with the corresponding prediction model as a function of the control parameters. The regression tables for the printing time (s), the part weight (g), the tensile strength (MPa), and EPC (MJ) are presented in Table 4, Table 5, Table 6 and Table 7, respectively. After each table, the corresponding prediction model is presented (Equations (7)–(10)). For each metric studied, the corresponding prediction model was established using regression analysis. This is an equation for the calculation of the specific metric as a function of the six control parameters studied herein. Such equations, when proven to be reliable, have industrial merit, as they can be applied to real application environments and estimate the expected value for the metric under investigation by giving as input the required values for the control parameters. For the remaining response metrics, the corresponding regression tables, and prediction models, are available in the supplementary material of the work.

For all response metrics, the F value was significantly higher than four (4) and the p value was almost zero (0). Regression values were higher than 85%, reaching values of about 95% in some cases, showing that the prediction models are sufficient for the response indicators in all cases evaluated herein.

(7) $$\mathrm{Printing}\;\mathrm{time}=42206-111.5\times \mathrm{ID}+1.514\times \mathrm{RDA}-225.5\times \mathrm{NT}-46.31\times \mathrm{PS}-10703\times \mathrm{LT}-153.7\times \mathrm{BT}\;\;\;\;\;\;\;\;\;\;\;\;+0.636\times {\mathrm{ID}}^{2}-0.01344\times {\mathrm{RDA}}^{2}+0.489\times {\mathrm{NT}}^{2}+0.3987\times {\mathrm{PS}}^{2}+19198\times {\mathrm{LT}}^{2}+0.704\times {\mathrm{BT}}^{2}$$

(8) $$\begin{array}{cc} \mathrm{Weight}=-57.09 & -0.2760\times \mathrm{ID}+0.005370\times \mathrm{RDA}+0.5031\times \mathrm{NT}-0.04358\times \mathrm{PS}-4.285\times \mathrm{LT}+0.2115\times \mathrm{BT}\\ & +0.001568\times {\mathrm{ID}}^{2}-0.000063\times {\mathrm{RDA}}^{2}-0.001053\times {\mathrm{NT}}^{2}+0.000440\times {\mathrm{PS}}^{2}+8.05\times {\mathrm{LT}}^{2}\\ & -0.000949\times {\mathrm{BT}}^{2} \end{array}$$

(9) $$\mathrm{sB}=1190-6.33\times \mathrm{ID}-0.2072\times \mathrm{RDA}-11.62\times \mathrm{NT}-2.201\times \mathrm{PS}+26.6\times \mathrm{LT}+8.12\times \mathrm{BT}+0.03545\times {\mathrm{ID}}^{2}\;\;\;\;\;\;\;-0.000270\times {\mathrm{RDA}}^{2}+0.02625\times {\mathrm{NT}}^{2}+0.02402\times {\mathrm{PS}}^{2}-158.2\times {\mathrm{LT}}^{2}-0.03622\times {\mathrm{BT}}^{2}$$

(10) $$\begin{array}{cc} \mathrm{EPC}=-0.93\;- & 0.0050\times \mathrm{ID}+0.000387\times \mathrm{RDA}+0.0181\times \mathrm{NT}-0.01624\times \mathrm{PS}-4.550\times \mathrm{LT}+0.0022\times \mathrm{BT}\\ & +0.000042\times {\mathrm{ID}}^{2}-0.000002\times {\mathrm{RDA}}^{2}-0.000042\times {\mathrm{NT}}^{2}+0.000144\times {\mathrm{PS}}^{2}+9.204\times {\mathrm{LT}}^{2}\\ & +0.000000\times {\mathrm{BT}}^{2} \end{array}$$

**Figure 11:**
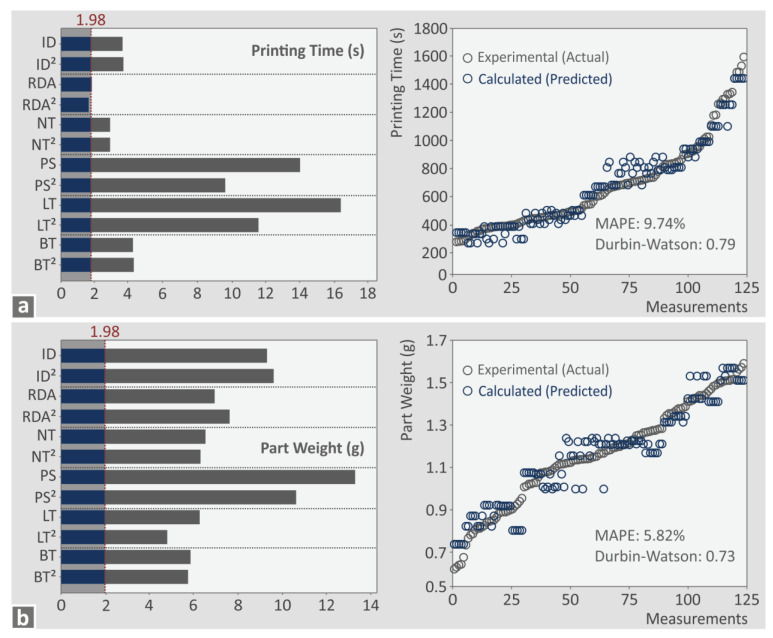
Pareto and experimental vs. calculated charts: (**a**) printing time (s), and (**b**) part weight (g).

**Figure 12:**
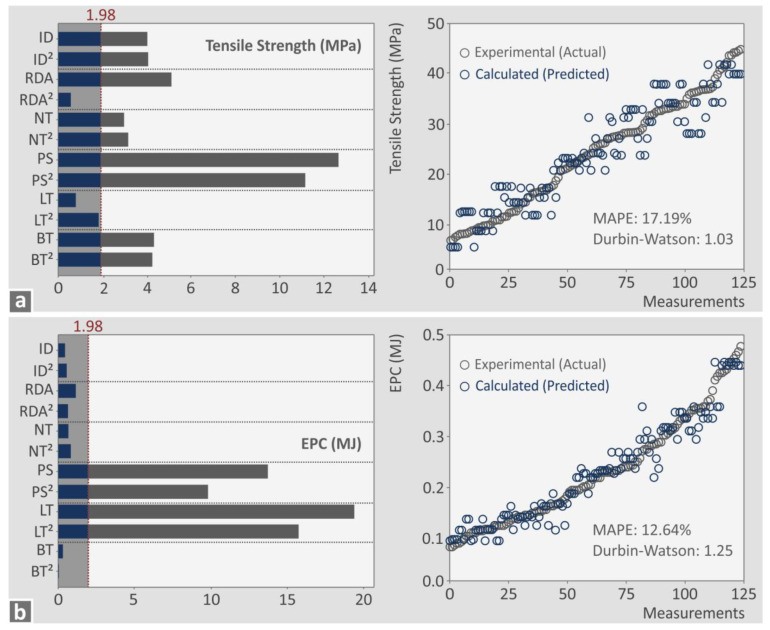
Pareto and experimental vs. calculated charts: (**a**) tensile strength (MPa), and (**b**) EPC (MJ).

Figure 11 and Figure 12 show Pareto charts created for the response metrics to identify the statistically important parameters for each metric. Next to each Pareto chart, a graph of the predicted vs. the actual metric values is shown. For each such graph, two metrics were calculated, the mean absolute percentage error (MAPE) (the prediction accuracy, <5% is acceptable) [91] and the Durbin-Watson factor (<2 means positive autocorrelation, equal to 2 means no autocorrelation, and >2 means negative autocorrelation) [92]. Pareto charts and predicted vs actual values graphs for additional response metrics can be found in the supplementary material of the work.

**Figure 13:**
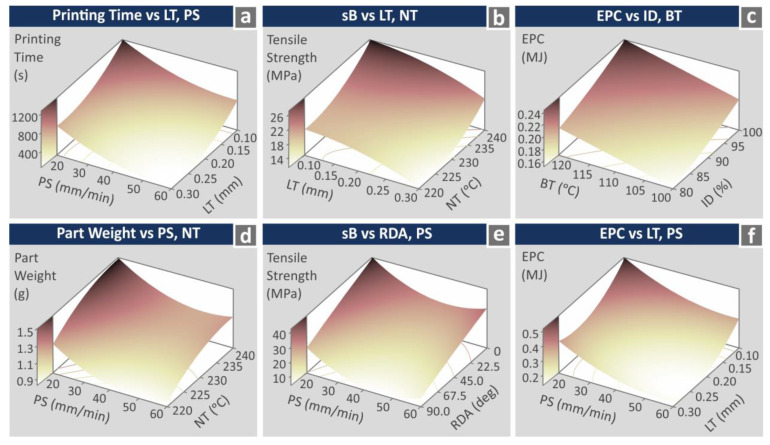
Three-dimensional surface graphs vs. the highest ranked control parameters for the response indicators: (**a**) printing time (s) vs. LT, PS, (**b**) tensile strength (MPa) vs. LT, NT, (**c**) EPC (MJ) vs. ID, BT, (**d**) part weight (g) vs. PS, NT, (**e**) sb (MPa) vs. RDA, PS, and (**f**) EPC (MJ) vs. LT, PS.

For the printing time (s), all parameters are statistically important (cross the 1.98 line), except RDA and RDA^2^ (Figure 11a). For the part weight (g), all parameters are statistically important (cross the 1.98 line) (Figure 11b). For both metrics, the MAPE was found to be higher than five (5), indicating reduced prediction accuracy; still, this is a quite good accuracy, and the result can be considered acceptable (less than 10, [93]). The corresponding Durbin–Watson factor for both metrics shows a positive autocorrelation of the residuals. For the tensile strength (MPa), all parameters are statistically important (cross the 1.98 line), except RDA^2^, LT, and LT^2^ (Figure 12a). For the EPC (MJ) metric, statistically important parameters (crossing the 1.98 line) were found to be PS, PS^2^, LT, and LT^2^. For both metrics, the MAPE was found to be higher than five (5), indicating reduced prediction accuracy. The corresponding Durbin–Watson factor for both metrics shows the positive autocorrelation of the residuals. Figure 13 presents printing time (s), part weight (g), tensile strength (MPa), and EPC (MJ) surface graphs against two of the higher ranked control parameters for each metric.

### 3.5. Confirmation Run

The control parameter values for the two confirmation runs performed (numbered run 26 and 27, respectively) are presented in Table 8. The average value and the deviation for each response metric studied herein in each one of the two confirmation runs are presented in Table 9 and Table 10. The analytical experimental results for each one of the five replicas in the confirmation runs can be found in the corresponding tables in the supplementary material of the work. The accuracy of the prediction models was verified by comparing the experimental values of the confirmation runs with the values calculated. The deviation (error) between those values for the tensile strength (MPa) and the EPC (MJ) metric for both confirmation runs is shown in Table 11. From the calculated absolute error, it is evident that the accuracy of the prediction models is more than sufficient.

## 4. Discussion

As shown in the literature review, 3D-printing settings significantly affect the performance of the parts built with the process. Herein, an attempt was made to produce parts with minimized energy requirements and optimized mechanical properties, with a polymer widely used in the specific type of applications, PMMA polymer, with the MEX 3D-printing process. The effect of six critical and machine-independent 3D-printing parameters was experimentally investigated and analyzed with modeling tools. Additionally, for the first time, insight is provided into the energy consumption when 3D printing PMMA parts with the MEX process. Such information affects the eco-friendliness of the process and increases its sustainability. It was found that energy and mechanical properties are not particularly controversial in this case, since the 3D-printing parameters that produced parts with the highest mechanical strength in the tensile tests required average amounts of energy, among the cases studied. Only in the case of the PS (mm/min) parameter are the results contradictory, as low PS values increase the tensile strength and the energy consumption. In this case, a value should be selected according to the requirements of each application and all the other 3D-printing settings should be adjusted accordingly.

Regarding the mechanical properties of the MEX 3D printing PMMA parts, the literature is still limited. Studies are focusing on the flexural, impact, and hardness properties of the parts made with PMMA in resin form [66,71]. Studies that have investigated the tensile properties of the 3D printed PMMA parts in the MEX [68] and the VPP process [65] are in good agreement with the results presented herein. The effect of the 3D printing parameters on the tensile properties of the build parts becomes evident by looking at the results of the work since, for example, the tensile strength of the parts built with the various parameters studied, varies from about 6 MPa to more than 40 MPa, which is more than 600% difference. Such variations in the results justify the need for the analysis conducted in the study, to comprehend the effect of the 3D printing settings on the build parts performance. The differences in the 3D printing settings affected the fracture mechanism on the samples, as it is shown in Figure 4. From the fracture surfaces it can be observed that in parts with less solid structure (more and larger voids shown in the fracture surface), such as the ones of run 4 and 5 for example, the mechanical performance was inferior to the parts with more solid structure, for example, the samples shown for runs 12 and 17. This is an expected response, as larger and more in number voids affect the mechanical performance of the parts, due to the reduced samples’ area and the inferior fusion between the strands [90].

Regarding energy consumption, there is no study available in the literature for the PMMA polymer in 3D printing. So, the results presented herein cannot be directly correlated to the literature. A study on the energy consumption of MEX 3D printed ABS parts [32] concluded that high PS (mm/min) and LT (mm) values significantly decrease the energy consumption for the manufacturing of the parts. These results agree with the findings presented herein. In [32] the mechanical properties of the ABS MEX 3D printed parts were also evaluated and the ID (%) was the dominant parameter for the tensile strength which is not the case here. The mechanical test findings agree regarding the effect of RDA and do not agree on the other 3D printing parameters. This difference can be attributed to the different materials studied mainly.

In the study presented herein, the 3D printing settings used during the process highly affected the energy requirements to build the parts, as the difference between the lowest and the highest EPC values recorded in the study is higher than 200 %. Such differences justify the need for the analysis conducted herein and also for the energy indicators. Such differences also indicate that with the proper selection of the 3D printing settings, the eco-friendliness and sustainability of the MEX 3D printing process can be significantly increased. Reduced energy demands also reduce the cost of the parts built with the process. The EPC values follow a similar pattern with the printing time and the part weight metrics, showing a strong correlation between them. For example, PS and LT were the two most important parameters for the printing time and the part weight. Their increase constantly decreased these two metrics up to the highest value studied. The same effect is shown for the EPC metric, with LT being the dominant parameter of the metric, as well. It should be noted though, that these findings refer to the specific 3D printer used and the specific control parameters and levels.

## 5. Conclusions

In this study, an attempt was made to evaluate the effect of six generic, machine-independent 3D-printing settings on the energy consumption and the tensile strength of parts built with the Poly[methyl methacrylate] polymer with material extrusion 3D printing. Such an approach has merit, especially in the case of the Poly[methyl methacrylate] polymer, for which the literature in material extrusion 3D printing is still rather limited. The experimental data were processed with statistical modeling tools and regression analysis. Raster deposition angle was the dominant parameter for the tensile strength, with low values achieving increased results. The layer thickness was the dominant parameter for energy consumption, with high values reducing the metric.

The values that minimized energy consumption (print speed of 60 mm/s and layer thickness of 0.25 mm) did not produce parts with increased tensile strength. On the other hand, a zero-degree raster deposition angle and nozzle temperature of 240 °C, which produced Poly[methyl methacrylate] parts with high tensile strength, required moderate energy amounts for the process. This indicates that a compromise should be made between the highest mechanical properties and the required energy for the production of the part. Still, these two criteria are not totally independent of each other.

To derive such results, the produced prediction models were evaluated with two confirmation runs with five replicas each, making the models acceptable for use in real-life applications. In future work, the applicability of the results can be expanded, by broadening the range of the control parameter values and by investigating additional 3D-printing settings.

## Figures and Tables

**Table 1:** Taguchi L25 Design: control parameters and levels.

Run	ID	RDA	NT	PS	LT	BT
1	80	0.0	220	20	0.10	100
2	80	22.5	225	30	0.15	105
3	80	45.0	230	40	0.20	110
4	80	67.5	235	50	0.25	115
5	80	90.0	240	60	0.30	120
6	85	0.0	225	40	0.25	120
7	85	22.5	230	50	0.30	100
8	85	45.0	235	60	0.10	105
9	85	67.5	240	20	0.15	110
10	85	90.0	220	30	0.20	115
11	90	0.0	230	60	0.15	115
12	90	22.5	235	20	0.20	120
13	90	45.0	240	30	0.25	100
14	90	67.5	220	40	0.30	105
15	90	90.0	225	50	0.10	110
16	95	0.0	235	30	0.30	110
17	95	22.5	240	40	0.10	115
18	95	45.0	220	50	0.15	120
19	95	67.5	225	60	0.20	100
20	95	90.0	230	20	0.25	105
21	100	0.0	240	50	0.20	105
22	100	22.5	220	60	0.25	110
23	100	45.0	225	20	0.30	115
24	100	67.5	230	30	0.10	120
25	100	90.0	235	40	0.15	100

**Table 2:** Average and standard deviation values of measured responses for weight, tensile strength (sB), tensile modulus of elasticity (E), and tensile toughness.

Run	Weight (g)	sB (MPa)	E (MPa)	Toughness (MJ/m^3^)
1	1.18 ± 0.04	40.85 ± 2.61	184.57 ± 4.25	7.75 ± 1.00
2	1.22 ± 0.02	31.25 ± 2.66	172.47 ± 7.34	3.68 ± 0.53
3	1.19 ± 0.01	21.20 ± 1.65	143.56 ± 8.04	1.63 ± 0.17
4	1.11 ± 0.01	15.23 ± 1.35	112.62 ± 8.15	0.84 ± 0.08
5	1.05 ± 0.03	14.93 ± 2.75	97.87 ± 15.79	1.09 ± 0.23
6	0.90 ± 0.02	27.55 ± 1.57	124.22 ± 4.06	4.10 ± 0.68
7	0.87 ± 0.00	12.11 ± 0.82	73.69 ± 3.69	1.27 ± 0.17
8	1.36 ± 0.01	27.39 ± 1.97	193.04 ± 7.37	2.19 ± 0.26
9	1.51 ± 0.00	35.64 ± 3.09	221.49 ± 12.00	2.99 ± 0.45
10	0.76 ± 0.05	8.71 ± 1.04	50.18 ± 4.78	1.09 ± 0.13
11	1.00 ± 0.01	32.78 ± 0.36	181.76 ± 4.71	3.72 ± 0.19
12	1.42 ± 0.01	44.23 ± 0.47	223.67 ± 1.03	5.46 ± 0.27
13	1.26 ± 0.01	25.00 ± 1.53	157.35 ± 10.87	2.02 ± 0.20
14	0.60 ± 0.02	6.53 ± 0.83	24.00 ± 4.25	0.42 ± 0.03
15	1.08 ± 0.02	8.68 ± 0.59	97.81 ± 13.91	0.47 ± 0.06
16	1.14 ± 0.03	27.97 ± 0.39	147.12 ± 4.59	3.51 ± 0.11
17	1.47 ± 0.02	37.79 ± 2.09	214.59 ± 5.49	4.31 ± 0.90
18	0.78 ± 0.03	10.48 ± 0.67	52.17 ± 1.72	1.56 ± 0.11
19	0.82 ± 0.02	7.31 ± 0.28	55.13 ± 3.44	0.71 ± 0.03
20	1.34 ± 0.02	21.66 ± 3.33	148.52 ± 11.89	1.72 ± 0.33
21	1.12 ± 0.01	32.61 ± 0.57	178.07 ± 7.21	4.06 ± 0.42
22	1.10 ± 0.04	35.81 ± 0.69	142.44 ± 6.29	6.78 ± 0.86
23	1.55 ± 0.04	28.38 ± 4.95	161.46 ± 22.72	3.62 ± 1.09
24	1.44 ± 0.04	20.96 ± 0.98	165.89 ± 4.49	1.54 ± 0.20
25	1.24 ± 0.04	15.34 ± 1.13	149.17 ± 14.70	0.54 ± 0.03

**Table 3:** Average and standard deviation values of measured responses for printing time, EPC, SPE, and SPP.

Run	Printing Time (s)	EPC (MJ)	SPE (MJ/g)	SPP (kW/g)
1	1522.20 ± 60.01	0.455 ± 0.023	0.386 ± 0.025	0.254 ± 0.013
2	684.80 ± 30.93	0.248 ± 0.020	0.204 ± 0.019	0.298 ± 0.041
3	470.40 ± 18.15	0.141 ± 0.005	0.119 ± 0.004	0.253 ± 0.009
4	375.40 ± 23.79	0.106 ± 0.018	0.095 ± 0.016	0.253 ± 0.035
5	254.60 ± 8.56	0.109 ± 0.032	0.104 ± 0.031	0.410 ± 0.135
6	372.20 ± 9.55	0.128 ± 0.009	0.142 ± 0.012	0.382 ± 0.037
7	326.80 ± 29.79	0.087 ± 0.012	0.101 ± 0.014	0.311 ± 0.056
8	686.00 ± 33.73	0.245 ± 0.022	0.179 ± 0.015	0.262 ± 0.025
9	1215.40 ± 86.92	0.340 ± 0.018	0.226 ± 0.012	0.186 ± 0.011
10	591.60 ± 11.76	0.225 ± 0.005	0.296 ± 0.023	0.500 ± 0.032
11	430.20 ± 16.28	0.186 ± 0.016	0.187 ± 0.017	0.433 ± 0.029
12	911.40 ± 24.35	0.316 ± 0.023	0.223 ± 0.015	0.244 ± 0.020
13	526.80 ± 11.86	0.149 ± 0.014	0.118 ± 0.011	0.224 ± 0.018
14	357.80 ± 10.71	0.163 ± 0.012	0.270 ± 0.022	0.754 ± 0.064
15	834.20 ± 13.90	0.315 ± 0.035	0.292 ± 0.031	0.351 ± 0.037
16	462.40 ± 21.00	0.216 ± 0.039	0.189 ± 0.030	0.408 ± 0.057
17	996.20 ± 30.77	0.360 ± 0.023	0.244 ± 0.016	0.245 ± 0.019
18	668.80 ± 22.20	0.238 ± 0.034	0.307 ± 0.051	0.458 ± 0.060
19	428.00 ± 11.34	0.147 ± 0.022	0.178 ± 0.025	0.417 ± 0.057
20	802.20 ± 27.45	0.298 ± 0.041	0.222 ± 0.029	0.278 ± 0.046
21	435.60 ± 18.99	0.115 ± 0.010	0.103 ± 0.009	0.237 ± 0.030
22	331.40 ± 21.95	0.112 ± 0.017	0.102 ± 0.019	0.308 ± 0.059
23	812.00 ± 105.91	0.369 ± 0.064	0.239 ± 0.045	0.296 ± 0.061
24	1313.80 ± 31.77	0.440 ± 0.019	0.305 ± 0.008	0.232 ± 0.008
25	691.20 ± 26.24	0.212 ± 0.014	0.170 ± 0.009	0.247 ± 0.020

**Table 4:** Polynomial ANOVA, printing time vs. ID, RDA, NT, PS, LT, BT.

Source	DF	Adj SS	Adj MS	F-Value	*p*-Value
Regression	12	12,606,678	10,50,557	181.50	0.000
ID	1	83,911	83,911	14.50	0.000
RDA	1	23,329	23,329	4.03	0.047
NT	1	52,548	52,548	9.08	0.003
PS	1	1,147,549	1,147,549	198.26	0.000
LT	1	1,532,669	1,532,669	264.80	0.000
BT	1	106,752	106,752	18.44	0.000
ID^2^	1	88,388	88,388	15.27	0.000
RDA^2^	1	16,198	16,198	2.80	0.097
NT^2^	1	52,387	52,387	9.05	0.003
PS^2^	1	556,406	556,406	96.13	0.000
LT^2^	1	806,208	806,208	139.29	0.000
BT^2^	1	108,451	108,451	18.74	0.000
Error	112	648,269	5788		
Total	124	13,254,947	1,056,345		
R^2^	95.11%				
R^2^ (adj)	94.59%				
R^2^ (pred)	93.96%				

**Table 5:** Polynomial ANOVA, weight vs. ID, RDA, NT, PS, LT, BT.

Source	DF	Adj SS	Adj MS	F-Value	*p*-Value
Regression	12	7.11585	0.59299	102.39	0
ID	1	0.51381	0.51381	88.72	0
RDA	1	0.29362	0.29362	50.70	0
NT	1	0.26159	0.26159	45.17	0
PS	1	1.01651	1.01651	175.52	0
LT	1	0.24564	0.24564	42.41	0
BT	1	0.20200	0.20200	34.88	0
ID^2^	1	0.53806	0.53806	92.91	0
RDA^2^	1	0.35444	0.35444	61.20	0
NT^2^	1	0.24257	0.24257	41.88	0
PS^2^	1	0.67637	0.67637	116.79	0
LT^2^	1	0.14169	0.14169	24.46	0
BT^2^	1	0.19688	0.19688	33.99	0
Error	112	0.64864	0.00579		
Total	124	7.76449	0.59878		
R^2^	91.65%				
R^2^ (adj)	90.75%				
R^2^ (pred)	89.59%				

**Table 6:** Polynomial ANOVA, sB vs. ID, RDA, NT, PS, LT, BT.

Source	DF	Adj SS	Adj MS	F-Value	*p*-Value
Regression	12	13,843.7	1153.64	72.57	0.000
ID	1	270.2	270.15	16.99	0.000
RDA	1	437.0	437.00	27.49	0.000
NT	1	139.5	139.52	8.78	0.004
PS	1	2593.5	2593.47	163.13	0.000
LT	1	9.5	9.46	0.60	0.442
BT	1	297.9	297.91	18.74	0.000
ID^2^	1	274.9	274.94	17.29	0.000
RDA^2^	1	6.6	6.56	0.41	0.522
NT^2^	1	150.7	150.73	9.48	0.003
PS^2^	1	2019.6	2019.56	127.03	0.000
LT^2^	1	54.8	54.76	3.44	0.066
BT^2^	1	286.9	286.92	18.05	0.000
Error	112	1780.6	15.90		
Total	124	15,624.3	1169.54		
R^2^	88.60%				
R^2^ (adj)	87.38%				
R^2^ (pred)	85.74%				

**Table 7:** Polynomial ANOVA, EPC vs. ID, RDA, NT, PS, LT, BT.

Source	DF	Adj SS	Adj MS	F-Value	*p*-Value
Regression	12	1.37912	0.114926	156.71	0.000
ID	1	0.00017	0.000171	0.23	0.630
RDA	1	0.00153	0.001526	2.08	0.152
NT	1	0.00034	0.000338	0.46	0.499
PS	1	0.14123	0.141228	192.58	0.000
LT	1	0.27703	0.277027	377.75	0.000
BT	1	0.00002	0.000021	0.03	0.866
ID^2^	1	0.00038	0.000378	0.52	0.474
RDA^2^	1	0.00053	0.000533	0.73	0.396
NT^2^	1	0.00039	0.000393	0.54	0.466
PS^2^	1	0.07216	0.072162	98.40	0.000
LT^2^	1	0.18530	0.185297	252.67	0.000
BT^2^	1	0.00000	0.000000	0.00	1.000
Error	112	0.08214	0.000733		
Total	124	1.46126	0.115659		
R^2^	94.38%				
R^2^ (adj)	93.78%				
R^2^ (pred)	92.95%				

**Table 8:** Control parameters for the confirmation runs.

Run	ID	RDA	NT	PS	LT	BT
26	100	0.0	240	20	0.10	112.1
27	80	0.0	240	56.8	0.25	100

**Table 9:** Average and standard deviation values of measured responses for weight, tensile strength, tensile modulus of elasticity, and tensile toughness, for the confirmation runs.

Run	Weight (g)	sB (MPa)	E (MPa)	Toughness (MJ/m^3^)
26	2.09 ± 0.07	65.06 ± 1.70	260.00 ± 10.71	8.62 ± 0.31
27	0.86 ± 0.01	28.20 ± 0.63	188.46 ± 5.67	3.88 ± 0.07

**Table 10:** Average and standard deviation values of measured responses for printing time, EPC, SPE, and SPP, for the confirmation runs.

Run	Printing Time (s)	EPC (MJ)	SPE (MJ/g)	SPP (kW/g)
26	1219.60 ± 45.99	0.600 ± 0.034	0.288 ± 0.024	0.236 ± 0.021
27	295.20 ± 10.18	0.069 ± 0.008	0.080 ± 0.008	0.272 ± 0.024

**Table 11:** Validation table.

Run		26	27
Actual	sB (MPa)	65.06	28.20
	EPC (MJ)	0.60	0.07
Predicted	sB (MPa)	56.46	32.83
	EPC (MJ)	0.53	0.06
Absolute Error	sB (%)	13.21	16.43
	EPC (%)	11.39	7.82

## Data Availability

The data presented in this study are available upon request from the corresponding author.

## Supplementary Materials

The following supporting information can be downloaded at: https://www.mdpi.com/article/10.3390/polym15040845/s1. Table S1. Measured Weight, Tensile Strength, Tensile Modulus of Elasticity and Tensile Toughness for each experimental run and five replicas per run; Table S2. Measured for Printing Time, EPC, SPE, SPP for each experimental run and five replicas per run; Table S3. Polynomial ANOVA, E vs ID, RDA, NT, PS, LT, BT; Table S4. Polynomial ANOVA, Toughness vs ID, RDA, NT, PS, LT, BT; Table S5. Polynomial ANOVA, SPE vs ID, RDA, NT, PS, LT, BT; Table S6. Polynomial ANOVA, SPP vs ID, RDA, NT, c, LT, BT; Table S7. Measured Weight, Tensile Strength, Tensile Modulus of Elasticity and Tensile Toughness for each experimental run and five replicas per run for the Confirmation Runs; Table S8. Measured Printing Time, EPC, SPE, SPP for each experimental run and five replicas per run for the Confirmation Runs; Figure S1. MEP: tensile modulus of elasticity (MPa), tensile toughness (MJ/m^3^); Figure S2. MEP: SPE (MJ/g), SPP (kW/g); Figure S3. Interaction plots: printing time (s), part weight (g); Figure S4. Interaction plots: tensile modulus of elasticity (MPa), tensile toughness (MJ/m^3^); Figure S5. Interaction plots: SPE (MJ/g), SPP (kW/g); Figure S6. Pareto and experimental vs calculated charts: (a) tensile modulus of elasticity (MPa), (b) tensile toughness (MJ/m^3^); Figure S7. Pareto and experimental vs calculated charts: (a) SPE (MJ/g), (b) SPP (kW/g).

## Nomenclature

3DP	3D printing
ABS	Acrylonitrile Butadiene Styrene
AM	Additive manufacturing
ANOVA	Analysis of variances
BT	Bed temperature
DF	Degrees of freedom
DOE	Design of experiment
DSC	Differential scanning calorimetry
E	Tensile modulus of elasticity
EPC	Energy printing consumption
FFF	Fused filament fabrication
ID	Infill density
LT	Layer thickness
MEP	Main effect plot
MEX	Material extrusion
NT	Nozzle temperature
ORA	Orientation angle
PA	Polyamide
PC	Polycarbonate
PMMA	Poly[methyl methacrylate]
PLA	Polylactic acid
PT	Printing time
PS	Printing speed
RDA	Raster deposition angle
QRM	Quadratic regression model
RQRM	Reduced quadratic regression model
sB	Compression strength
SEM	Scanning electron microscopy
SPE	Specific printing energy
SPP	Specific printing power
Tg	Glass transition temperature
TGA	Thermogravimetric analysis
