# Supplementary material for: Energy Consumption vs. Tensile Strength of Poly[methyl methacrylate] in Material Extrusion 3D Printing: The Impact of Six Control Settings
Source: Polymers (Basel). 2023 Feb 8;15(4):845. doi: 10.3390/polym15040845 (PMC9966017; doi:10.3390/polym15040845)
Supplement: Supplementary file 1 [file polymers-15-00845-s001.zip › polymers-2206707-supplementary.pdf]

Supplementary Material for Article

Energy Consumption vs. Tensile Strength of Poly[methyl methacrylate] in Material Extrusion 3D Printing: The Impact of Six Control Settings

Nectarios Vidakis [1], Markos Petousis [1,*], Nikolaos Mountakis [1], Amalia Moutsopoulou [1] and Emmanuel Karapidakis [2]

[1] Department of Mechanical Engineering, Hellenic Mediterranean University, 71410 Heraklion, Greece
[2] Electrical and Computer Engineering Department, Hellenic Mediterranean University, 71410 Heraklion, Greece
* Correspondence: markospetousis@hmu.gr; Tel.: +30-281-037-9227

Abstract: The energy efficiency of material extrusion additive manufacturing has a significant impact on the economics and environmental footprint of the process. Control parameters that ensure 3D-printed functional products of premium quality and mechanical strength are an established market-driven requirement. To accomplish multiple objectives is challenging, especially for multi-purpose industrial polymers, such as the Poly[methyl methacrylate]. The current paper explores the contribution of six generic control factors (infill density, raster deposition angle, nozzle temperature, print speed, layer thickness, and bed temperature) to the energy performance of Poly[methyl methacrylate] over its mechanical performance. A five-level L25 Taguchi orthogonal array was composed, with five replicas, involving 135 experiments. The 3D printing time and the electrical consumption were documented with the stopwatch approach. The tensile strength, modulus, and toughness were experimentally obtained. The raster deposition angle and the printing speed were the first and second most influential control parameters on tensile strength. Layer thickness and printing speed were the corresponding ones for the energy consumption. Quadratic regression model equations for each response metric over the six control parameters were compiled and validated. Thus, the best compromise between energy efficiency and mechanical strength is achievable, and a tool creates significant value for engineering applications.

Keywords: Poly[methyl methacrylate] (PMMA); optimization; material extrusion (MEX); energy consumption; energy efficiency; compressive strength; taguchi analysis; robust design

Citation: Vidakis, N.; Petousis, M.; Mountakis, N.; Moutsopoulou, A.; Karapidakis, E. Energy Consumption vs. Tensile Strength of Poly[methyl methacrylate] in Material Extrusion 3D Printing: The Impact of Six Control Settings. *Polymers* **2023**, *15*, 845. https://doi.org/10.3390/polym15040845

Academic Editor: Filippo Berto

Received: 23 January 2023
Revised: 5 February 2023
Accepted: 6 February 2023
Published: 8 February 2023

Copyright: © 2023 by the authors. Licensee MDPI, Basel, Switzerland. This article is an open access article distributed under the terms and conditions of the Creative Commons Attribution (CC BY) license (https://creativecommons.org/licenses/by/4.0/).

3. Results

3.2. DOE and experimental results

Table S1. Measured Weight, Tensile Strength, Tensile Modulus of Elasticity and Tensile Toughness for each experimental run and five replicas per run.

A/A	Run	Weight (g)	sB (MPa)	E (MPa)	Toughness (MJ/m^3)
1		1.255	36.65	183.90	6.32
2		1.149	43.62	184.33	8.86
3	1	1.178	41.30	190.58	7.25
4		1.149	42.13	185.36	7.88
5		1.164	40.54	178.65	8.43
6		1.196	33.15	173.18	4.30
7		1.239	26.86	159.74	2.96
8	2	1.239	33.59	176.40	4.07
9		1.215	31.35	175.00	3.48
10		1.220	31.28	178.03	3.60
11		1.182	21.97	148.50	1.79
12		1.201	23.10	149.70	1.78
13	3	1.167	18.63	129.72	1.37
14		1.196	21.42	144.22	1.62
15		1.191	20.88	145.67	1.60
16		1.120	13.18	101.68	0.74
17		1.124	15.80	118.43	0.87
18	4	1.105	16.53	122.34	0.95
19		1.096	16.02	108.39	0.78
20		1.115	14.59	112.27	0.89
21		1.034	10.58	78.69	0.77
22		1.006	14.40	102.11	1.11
23	5	1.063	17.97	120.70	1.40
24		1.097	15.97	88.45	1.16
25		1.054	15.73	99.38	1.03
26		0.915	29.77	126.54	4.94
27		0.891	25.53	121.00	3.13
28	6	0.877	27.20	124.52	4.40
29		0.930	28.19	129.53	3.79
30		0.901	27.07	119.52	4.25
31		0.862	11.71	68.46	1.17
32		0.872	13.44	75.16	1.54
33	7	0.862	11.37	75.60	1.08
34		0.867	12.37	71.49	1.31
35		0.862	11.68	77.74	1.24
36		1.359	30.35	192.17	2.60
37		1.349	27.19	201.03	2.10
38	8	1.378	25.03	181.83	1.91
39		1.368	26.44	198.13	2.11
40		1.368	27.96	192.04	2.20
41		1.511	31.66	224.96	2.66
42	9	1.511	33.57	208.44	2.76
43		1.506	39.64	235.17	3.77

44		1.501	36.77	209.49	2.89
45		1.506	36.58	229.41	2.88
46		0.700	9.92	53.62	1.25
47		0.734	7.05	41.76	0.88
48	10	0.782	8.94	51.49	1.07
49		0.820	8.90	52.07	1.13
50		0.782	8.74	51.97	1.13
51		0.990	32.23	175.72	3.86
52		0.985	33.11	181.64	3.56
53	11	1.000	33.10	184.58	3.48
54		1.005	32.81	179.04	3.93
55		1.000	32.68	187.82	3.74
56		1.404	44.80	224.60	5.77
57		1.433	44.51	223.60	5.29
58	12	1.433	43.54	222.82	5.18
59		1.409	44.13	224.82	5.32
60		1.419	44.20	222.51	5.72
61		1.252	25.17	163.85	1.95
62		1.257	22.72	150.20	1.92
63	13	1.262	26.95	171.45	2.39
64		1.266	25.51	157.35	1.94
65		1.262	24.63	143.92	1.92
66		0.582	5.92	21.10	0.40
67		0.640	5.75	20.15	0.39
68	14	0.606	7.83	30.71	0.47
69		0.592	6.41	25.42	0.40
70		0.602	6.73	22.60	0.42
71		1.098	8.45	90.59	0.39
72		1.088	9.64	118.27	0.50
73	15	1.055	8.09	80.63	0.54
74		1.059	8.79	100.00	0.45
75		1.079	8.41	99.58	0.49
76		1.102	27.71	142.48	3.49
77		1.135	28.66	149.07	3.40
78	16	1.178	27.81	142.00	3.45
79		1.164	27.88	149.99	3.69
80		1.130	27.82	152.07	3.51
81		1.454	35.97	214.49	3.76
82		1.492	36.13	207.85	3.48
83	17	1.483	40.92	214.98	5.70
84		1.464	37.10	223.01	3.88
85		1.478	38.83	212.63	4.71
86	18	0.822	9.80	50.15	1.48
87		0.774	9.93	50.69	1.55

88		0.750	11.48	54.26	1.75
89		0.755	10.51	52.63	1.54
90		0.789	10.68	53.13	1.48
91		0.797	7.06	50.13	0.76
92		0.811	7.73	59.11	0.71
93	19	0.854	7.12	56.96	0.67
94		0.835	7.47	53.54	0.71
95		0.816	7.17	55.91	0.71
96		1.320	22.10	145.48	1.70
97		1.373	17.05	134.30	1.20
98	20	1.344	25.69	167.04	2.10
99		1.344	23.56	150.15	1.74
100		1.335	19.89	145.61	1.84
101		1.132	32.23	183.25	3.77
102		1.122	32.09	177.46	3.66
103	21	1.122	33.54	169.16	4.73
104		1.103	32.67	187.03	3.99
105		1.117	32.53	173.48	4.17
106		1.150	36.26	149.67	7.28
107		1.059	36.53	136.94	7.83
108	22	1.069	34.75	144.62	5.63
109		1.121	35.58	134.86	6.27
110		1.117	35.92	146.11	6.90
111		1.494	23.23	126.26	2.89
112		1.547	36.59	184.92	5.52
113	23	1.590	27.81	174.41	3.01
114		1.571	26.59	153.57	3.19
115		1.561	27.66	168.13	3.47
116		1.505	20.33	172.72	1.37
117		1.433	20.16	164.36	1.37
118	24	1.404	22.58	160.29	1.83
119		1.423	20.56	166.47	1.63
120		1.442	21.18	165.62	1.49
121		1.183	16.70	162.42	0.58
122		1.250	13.56	127.47	0.49
123	25	1.270	15.52	163.35	0.55
124		1.270	15.34	145.70	0.52
125		1.236	15.59	146.89	0.55
	Min:	0.582	5.75	20.15	0.39
	Max:	1.590	44.80	235.17	8.86
	Average:	1.141	23.62	138.91	2.68

Table S2. Measured for Printing Time, EPC, SPE, SPP for each experimental run and five replicas per run.

A/A	Run	Printing Time (s)	EPC (MJ)	SPE (MJ/g)	SPP (kW/g)
1	1	1546	0.457	0.364	0.236
2		1453	0.428	0.373	0.257
3		1501	0.436	0.370	0.246
4		1612	0.482	0.420	0.260
5		1499	0.472	0.405	0.270
6	2	642	0.284	0.238	0.370
7		692	0.238	0.192	0.277
8		668	0.241	0.195	0.291
9		723	0.241	0.199	0.275
10		699	0.238	0.195	0.279
11	3	471	0.148	0.125	0.265
12		494	0.144	0.120	0.243
13		477	0.137	0.117	0.246
14		444	0.137	0.114	0.258
15		466	0.140	0.118	0.253
16	4	394	0.104	0.093	0.237
17		402	0.119	0.106	0.263
18		357	0.112	0.101	0.283
19		346	0.076	0.069	0.199
20		378	0.119	0.107	0.282
21	5	247	0.162	0.157	0.634
22		255	0.086	0.086	0.337
23		252	0.108	0.102	0.403
24		269	0.083	0.075	0.281
25		250	0.104	0.099	0.396
26	6	365	0.119	0.130	0.356
27		387	0.126	0.141	0.365
28		363	0.140	0.160	0.441
29		371	0.122	0.132	0.355
30		375	0.133	0.148	0.394
31	7	348	0.079	0.092	0.264
32		364	0.094	0.107	0.295
33		297	0.104	0.121	0.408
34		298	0.076	0.087	0.293
35		327	0.083	0.096	0.294
36	8	718	0.238	0.175	0.244
37		632	0.248	0.184	0.291
38		708	0.281	0.204	0.288
39		678	0.227	0.166	0.245
40		694	0.230	0.168	0.243
41	9	1264	0.331	0.219	0.173
42		1337	0.360	0.238	0.178

43		1111	0.317	0.210	0.189
44		1181	0.356	0.237	0.201
45		1184	0.335	0.222	0.188
46		611	0.227	0.324	0.530
47		587	0.227	0.309	0.526
48	10	584	0.230	0.295	0.505
49		582	0.216	0.263	0.453
50		594	0.227	0.290	0.488
51		449	0.191	0.193	0.429
52		420	0.187	0.190	0.453
53	11	444	0.202	0.202	0.454
54		410	0.158	0.158	0.384
55		428	0.191	0.191	0.446
56		951	0.306	0.218	0.229
57		915	0.302	0.211	0.231
58	12	895	0.356	0.249	0.278
59		889	0.302	0.215	0.241
60		907	0.313	0.221	0.243
61		526	0.130	0.104	0.197
62		516	0.151	0.120	0.233
63	13	523	0.144	0.114	0.218
64		547	0.169	0.134	0.244
65		522	0.151	0.120	0.230
66		345	0.148	0.254	0.735
67		373	0.158	0.248	0.664
68	14	351	0.162	0.267	0.762
69		362	0.180	0.304	0.840
70		358	0.166	0.275	0.768
71		826	0.353	0.321	0.389
72		838	0.288	0.265	0.316
73	15	817	0.274	0.259	0.317
74		836	0.346	0.326	0.390
75		854	0.313	0.290	0.340
76		483	0.191	0.173	0.358
77		437	0.191	0.168	0.385
78	16	478	0.284	0.241	0.505
79		471	0.212	0.182	0.387
80		443	0.202	0.178	0.403
81		951	0.371	0.255	0.268
82		979	0.360	0.241	0.246
83	17	1022	0.338	0.228	0.223
84		1007	0.338	0.231	0.230
85		1022	0.392	0.265	0.260
86	18	653	0.227	0.276	0.423

87		650	0.194	0.251	0.386
88		681	0.248	0.331	0.486
89		702	0.288	0.381	0.543
90		658	0.234	0.297	0.451
91		430	0.115	0.145	0.336
92		427	0.148	0.182	0.426
93	19	417	0.140	0.164	0.394
94		420	0.173	0.207	0.493
95		446	0.158	0.194	0.435
96		767	0.324	0.245	0.320
97		778	0.356	0.260	0.334
98	20	821	0.274	0.204	0.248
99		820	0.256	0.190	0.232
100		825	0.281	0.210	0.255
101		441	0.108	0.095	0.216
102		411	0.133	0.119	0.289
103	21	421	0.112	0.099	0.236
104		449	0.112	0.101	0.225
105		456	0.112	0.100	0.219
106		314	0.097	0.085	0.269
107		328	0.137	0.129	0.394
108	22	341	0.119	0.111	0.326
109		364	0.097	0.087	0.238
110		310	0.108	0.097	0.312
111		936	0.428	0.287	0.306
112		899	0.374	0.242	0.269
113	23	742	0.270	0.170	0.229
114		682	0.421	0.268	0.393
115		801	0.353	0.226	0.282
116		1303	0.464	0.309	0.237
117		1352	0.446	0.312	0.230
118	24	1340	0.414	0.295	0.220
119		1300	0.443	0.311	0.239
120		1274	0.432	0.300	0.235
121		721	0.198	0.167	0.232
122		713	0.198	0.158	0.222
123	25	668	0.216	0.170	0.255
124		662	0.230	0.181	0.274
125		692	0.216	0.175	0.253
	Min:	247	0.076	0.069	0.173
	Max:	1612	0.482	0.420	0.840
	Average:	660.06	0.229	0.200	0.330

3.3. Statistical analysis

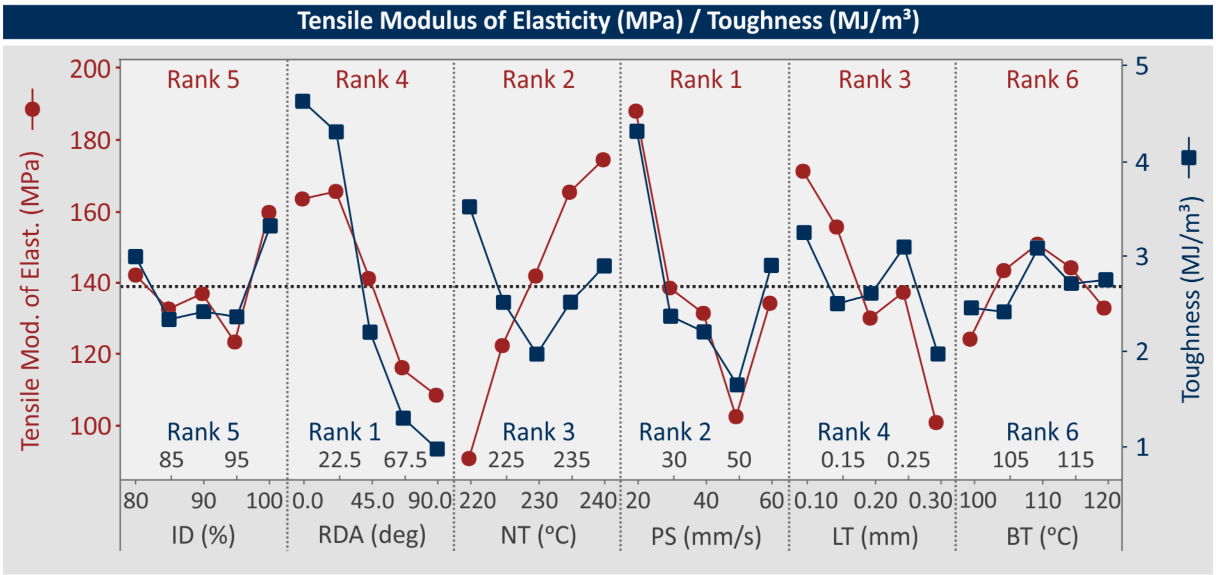

Figure S1. MEP: tensile modulus of elasticity (MPa), tensile toughness (MJ/m³)

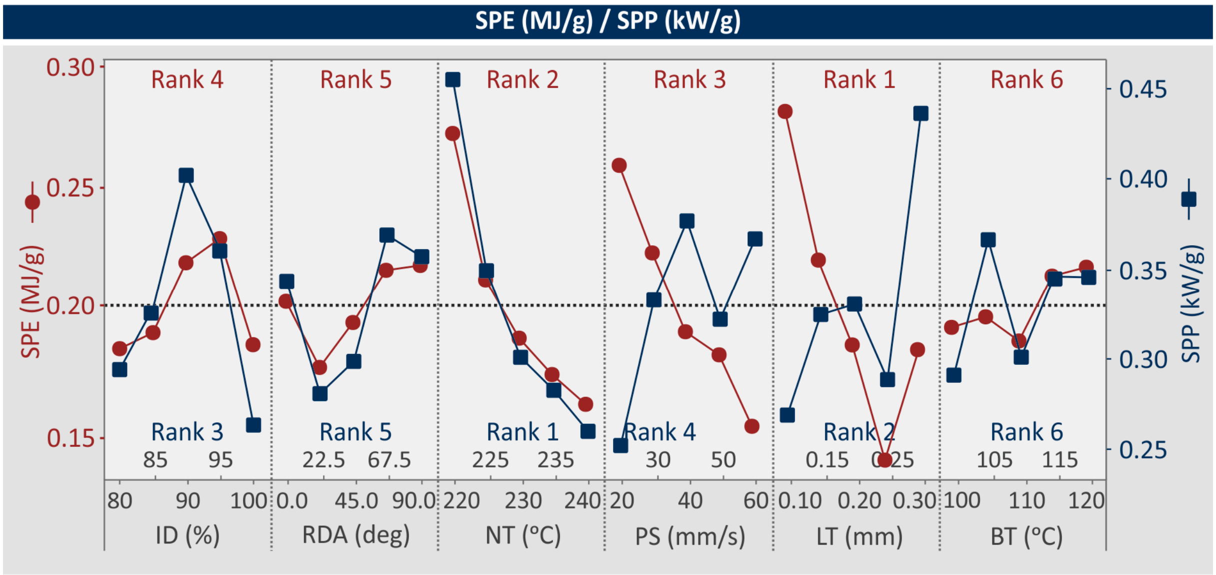

Figure S2. MEP: SPE (MJ/g), SPP (kW/g)

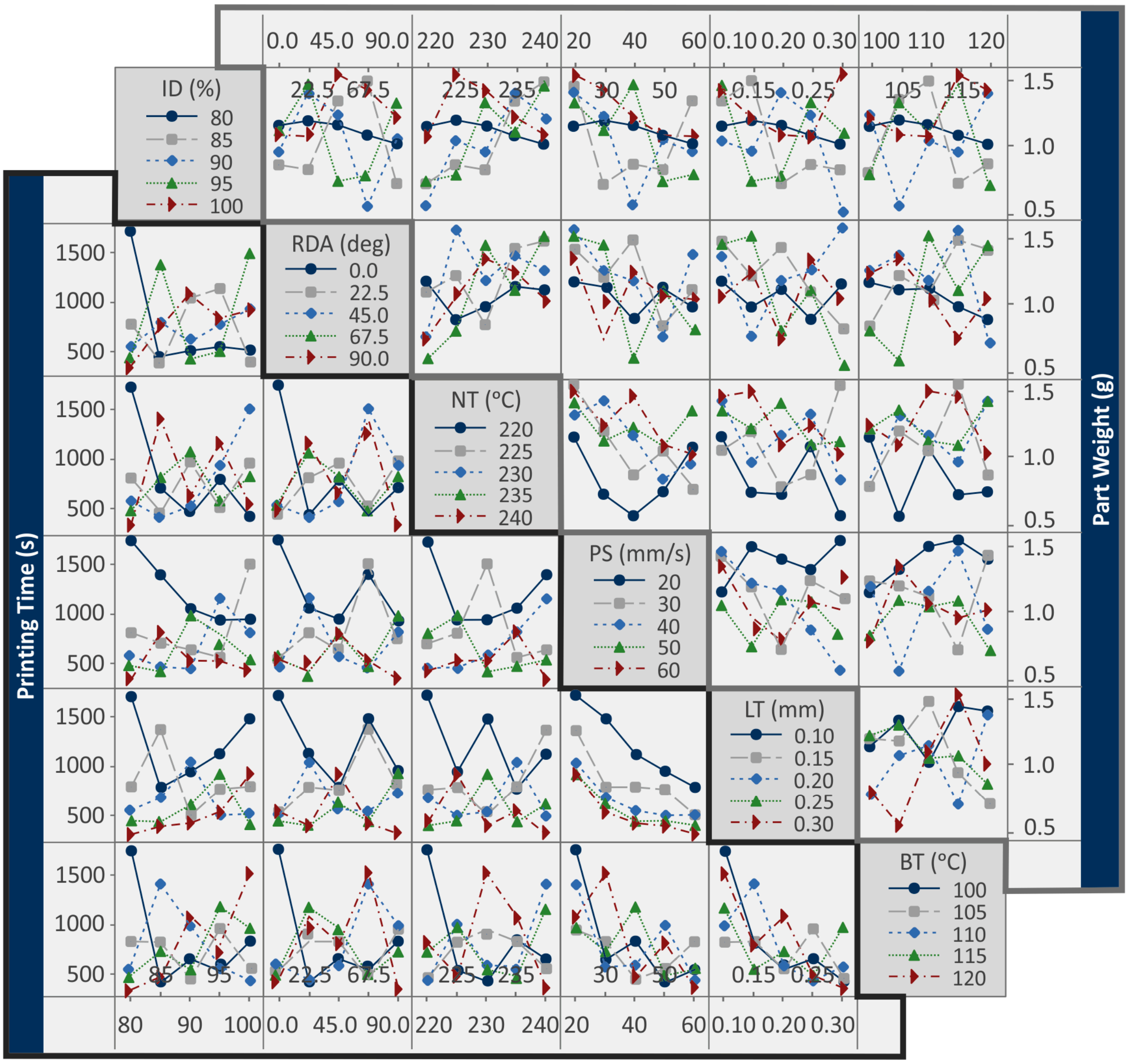

Figure S3. Interaction plots: printing time (s), part weight (g)

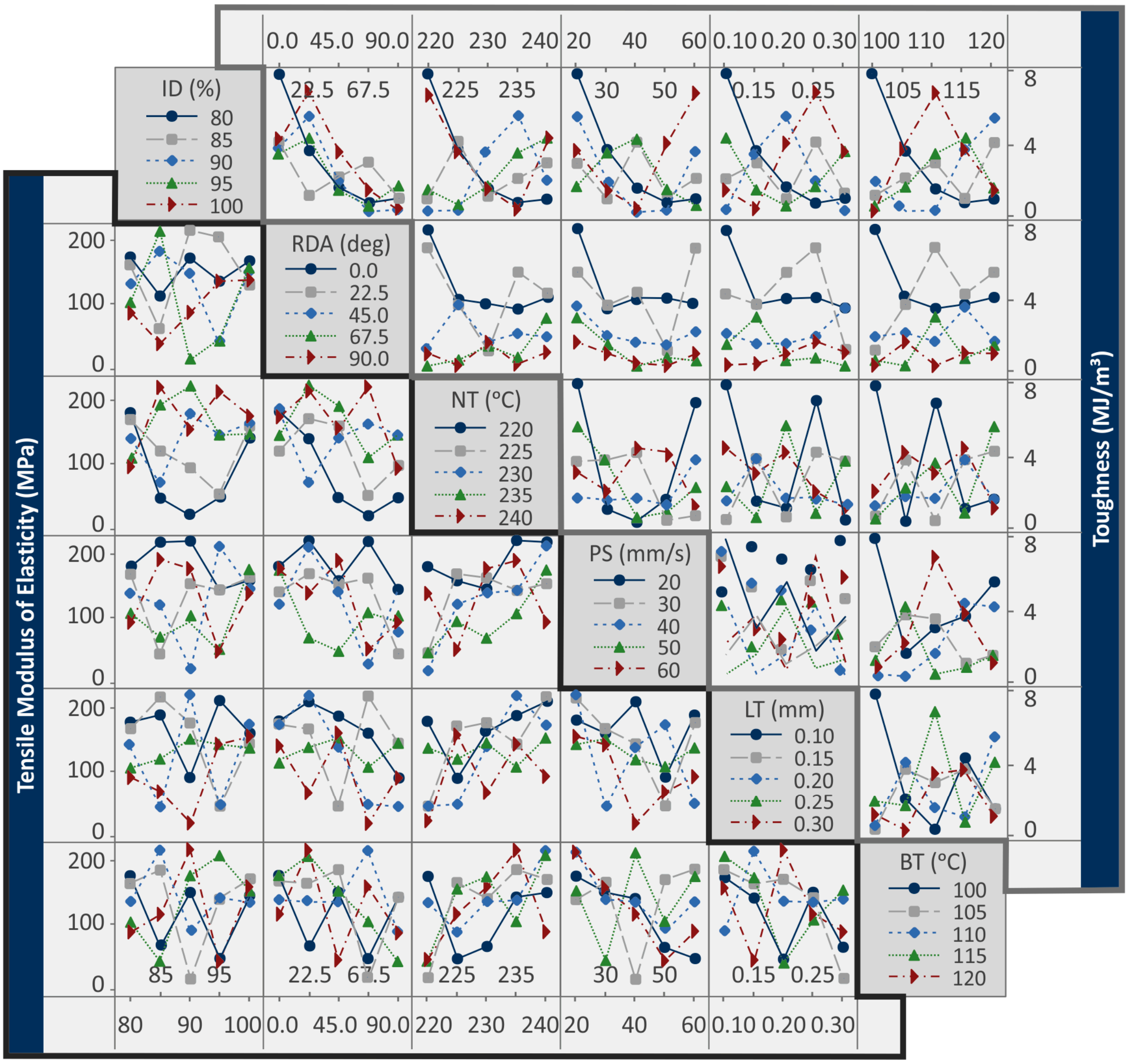

Figure S4. Interaction plots: tensile modulus of elasticity (MPa), tensile toughness (MJ/m³)

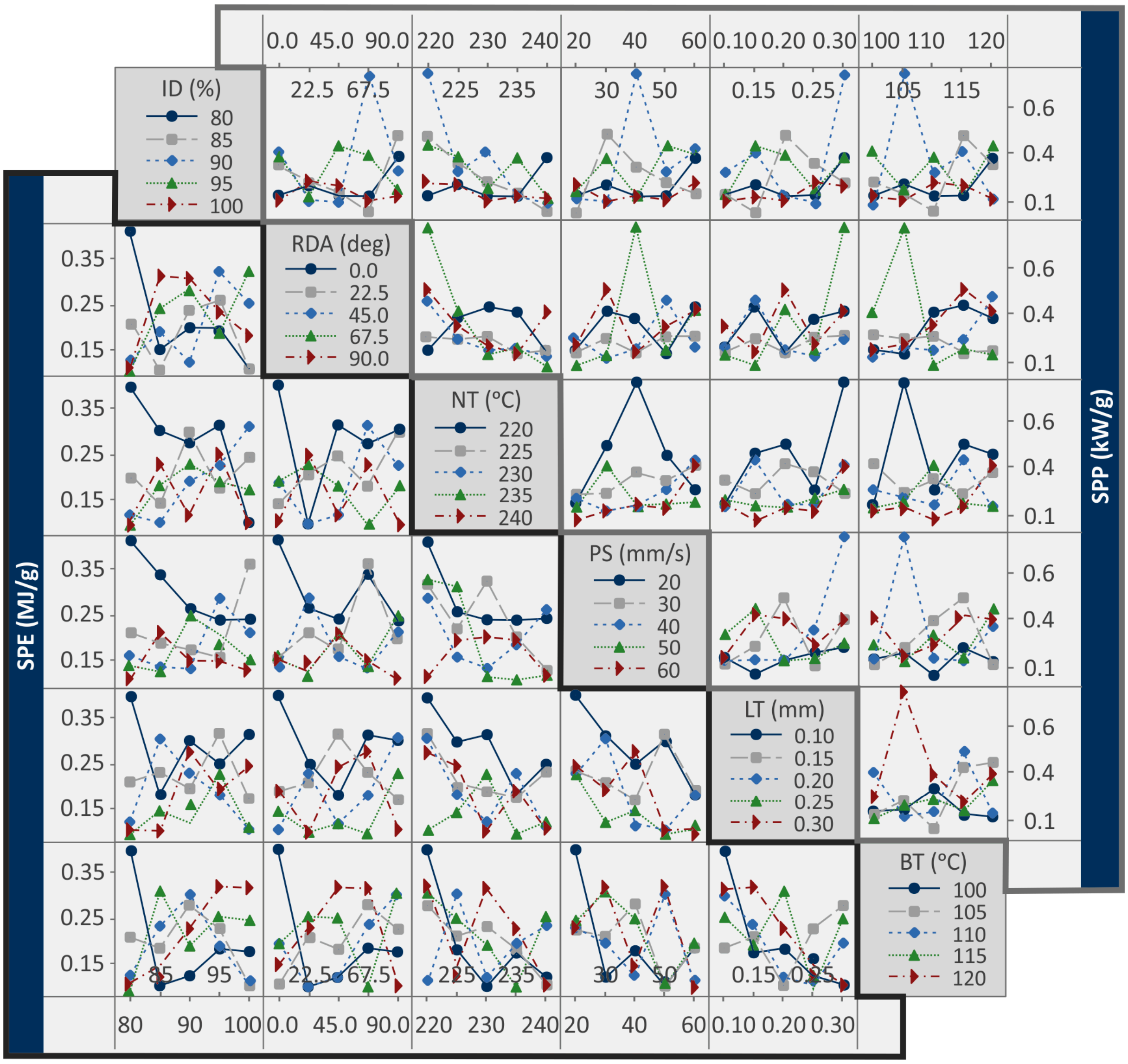

Figure S5. Interaction plots: SPE (MJ/g), SPP (kW/g)

3.4. Regression analysis

Table S3. Polynomial ANOVA, E vs ID, RDA, NT, PS, LT, BT.

Source	DF	Adj SS	Adj MS	F-Value	P-Value
Regression	12	348540	29045.0	89.73	0.000
ID	1	9348	9347.7	28.88	0.000
RDA	1	2004	2003.6	6.19	0.014
NT	1	3911	3910.7	12.08	0.001
PS	1	47806	47805.6	147.69	0.000
LT	1	642	641.8	1.98	0.162
BT	1	10148	10147.9	31.35	0.000
ID^2	1	9614	9614.5	29.70	0.000
RDA^2	1	752	751.7	2.32	0.130
NT^2	1	3397	3397.1	10.50	0.002
PS^2	1	35142	35141.8	108.57	0.000

LT^2	1	134	134.4	0.42	0.521
BT^2	1	9998	9997.6	30.89	0.000
Error	112	36253	323.7		
Total	124	384793	29368.7		
R^2	90.58%				
R^2 (adj)	89.57%				
R^2 (pred)	88.22%				

$$\begin{aligned} E = -8103 - 37.23 \times ID - 0.444 \times RDA + 61.5 \times NT - 9.451 \times PS - 219 \times LT + 47.40 \times BT \\ + 0.2096 \times ID^2 - 0.00289 \times RDA^2 - 0.1246 \times NT^2 + 0.10020 \times PS^2 \\ - 248 \times LT^2 - 0.2138 \times BT^2 \end{aligned} \quad (S1)$$

Table S4. Polynomial ANOVA, Toughness vs ID, RDA, NT, PS, LT, BT.

Source	DF	Adj SS	Adj MS	F-Value	P-Value
Regression	12	424.274	35.3562	56.35	0.000
ID	1	16.770	16.7701	26.73	0.000
RDA	1	38.002	38.0020	60.57	0.000
NT	1	26.839	26.8391	42.78	0.000
PS	1	76.565	76.5647	122.03	0.000
LT	1	0.001	0.0013	0.00	0.964
BT	1	1.430	1.4297	2.28	0.134
ID^2	1	17.065	17.0651	27.20	0.000
RDA^2	1	2.620	2.6197	4.18	0.043
NT^2	1	26.579	26.5795	42.36	0.000
PS^2	1	64.273	64.2731	102.44	0.000
LT^2	1	0.176	0.1756	0.28	0.598
BT^2	1	1.342	1.3418	2.14	0.146
Error	112	70.269	0.6274		
Total	124	494.543	35.9836		
R^2	85.79%				
R^2 (adj)	84.27%				
R^2 (pred)	82.08%				

$$\begin{aligned} Toughness = 639.3 - 1.577 \times ID - 0.06109 \times RDA - 5.096 \times NT - 0.3782 \times PS - 0.31 \times LT \\ + 0.563 \times BT + 0.00883 \times ID^2 + 0.000171 \times RDA^2 + 0.01102 \times NT^2 \\ + 0.004285 \times PS^2 - 9.0 \times LT^2 - 0.00248 \times BT^2 \end{aligned} \quad (S2)$$

Table S5. Polynomial ANOVA, SPE vs ID, RDA, NT, PS, LT, BT.

Source	DF	Adj SS	Adj MS	F-Value	P-Value
Regression	12	0.710921	0.059243	62.02	0.000
ID	1	0.027352	0.027352	28.63	0.000
RDA	1	0.002293	0.002293	2.40	0.124
NT	1	0.021942	0.021942	22.97	0.000
PS	1	0.012143	0.012143	12.71	0.001
LT	1	0.110442	0.110442	115.62	0.000

BT	1	0.002001	0.002001	2.09	0.151
ID^2	1	0.026635	0.026635	27.88	0.000
RDA^2	1	0.006938	0.006938	7.26	0.008
NT^2	1	0.020370	0.020370	21.32	0.000
PS^2	1	0.002542	0.002542	2.66	0.106
LT^2	1	0.072472	0.072472	75.87	0.000
BT^2	1	0.002267	0.002267	2.37	0.126
Error	112	0.106989	0.000955		
Total	124	0.817910	0.060198		
R^2	86.92%				
R^2 (adj)	85.52%				
R^2 (pred)	83.64%				

$$\begin{aligned}\mathrm{SPE} = 16.20 &+ 0.0637 \times \mathrm{ID} - 0.000475 \times \mathrm{RDA} - 0.1457 \times \mathrm{NT} - 0.00476 \times \mathrm{PS} - 2.873 \times \mathrm{LT} \\ &- 0.0210 \times \mathrm{BT} - 0.000349 \times \mathrm{ID}^2 + 0.000009 \times \mathrm{RDA}^2 + 0.000305 \times \mathrm{NT}^2 \\ &+ 0.000027 \times \mathrm{PS}^2 + 5.756 \times \mathrm{LT}^2 + 0.000102 \times \mathrm{BT}^2\end{aligned} \tag{S3}$$

Table S6. Polynomial ANOVA, SPP vs ID, RDA, NT, PS, LT, BT.

Source	DF	Adj SS	Adj MS	F-Value	P-Value
Regression	12	1.36387	0.113656	19.57	0.000
ID	1	0.25149	0.251490	43.31	0.000
RDA	1	0.02083	0.020835	3.59	0.061
NT	1	0.07384	0.073843	12.72	0.001
PS	1	0.07698	0.076977	13.26	0.000
LT	1	0.01191	0.011909	2.05	0.155
BT	1	0.00347	0.003467	0.60	0.441
ID^2	1	0.25313	0.253129	43.59	0.000
RDA^2	1	0.04188	0.041876	7.21	0.008
NT^2	1	0.06892	0.068921	11.87	0.001
PS^2	1	0.05266	0.052664	9.07	0.003
LT^2	1	0.03241	0.032406	5.58	0.020
BT^2	1	0.00304	0.003036	0.52	0.471
Error	112	0.65034	0.005807		
Total	124	2.01421	0.119463		
R^2	67.71%				
R^2 (adj)	64.25%				
R^2 (pred)	59.30%				

$$\begin{aligned}\mathrm{SPP} = 21.61 &+ 0.1931 \times \mathrm{ID} - 0.001430 \times \mathrm{RDA} - 0.2673 \times \mathrm{NT} + 0.01199 \times \mathrm{PS} - 0.943 \times \mathrm{LT} \\ &+ 0.0277 \times \mathrm{BT} - 0.001076 \times \mathrm{ID}^2 + 0.000022 \times \mathrm{RDA}^2 + 0.000561 \times \mathrm{NT}^2 \\ &- 0.000123 \times \mathrm{PS}^2 + 3.85 \times \mathrm{LT}^2 - 0.000118 \times \mathrm{BT}^2\end{aligned} \tag{S4}$$

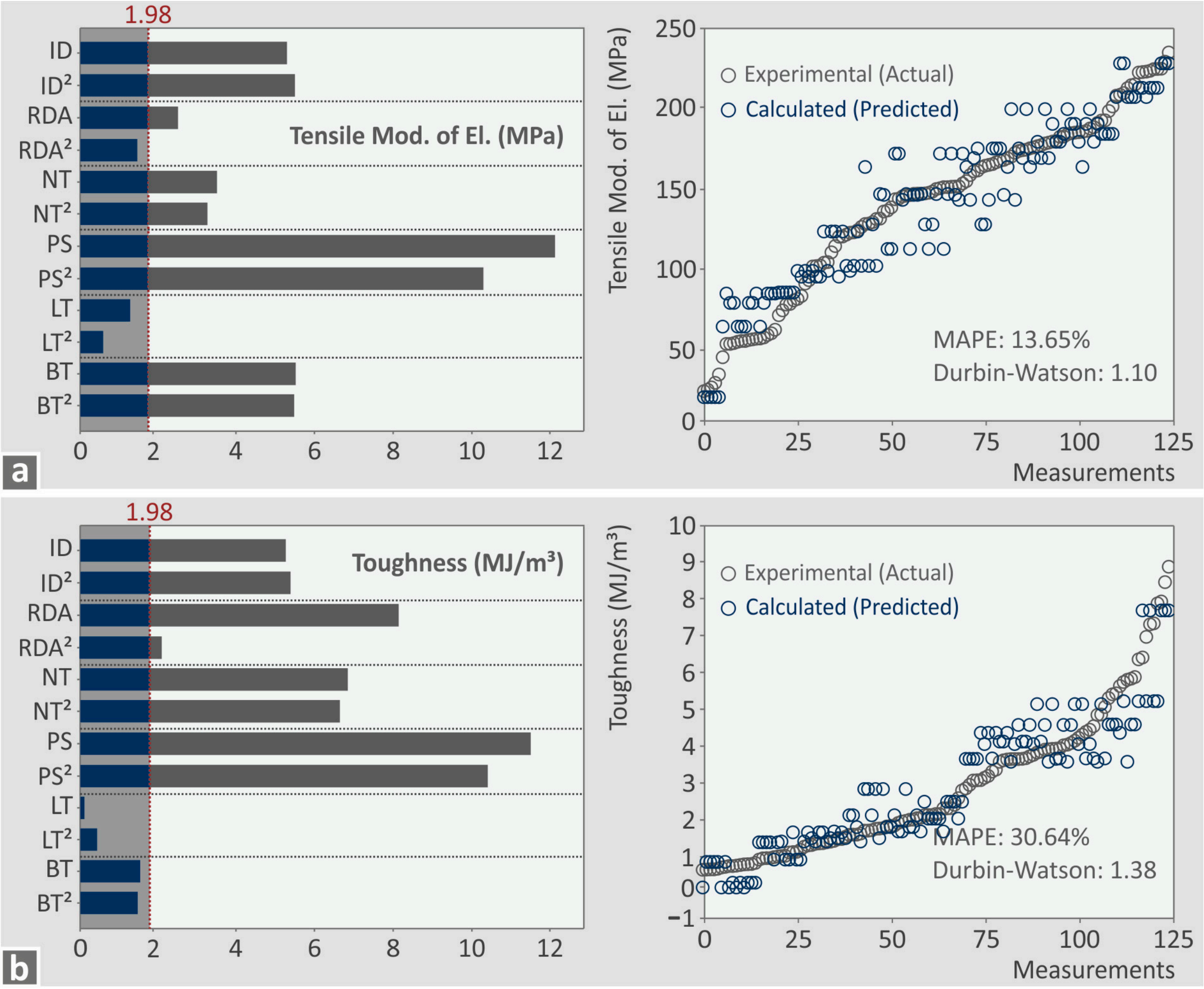

Figure S6. Pareto and experimental vs calculated charts: (a) tensile modulus of elasticity (MPa), (b) tensile toughness (MJ/m^3)

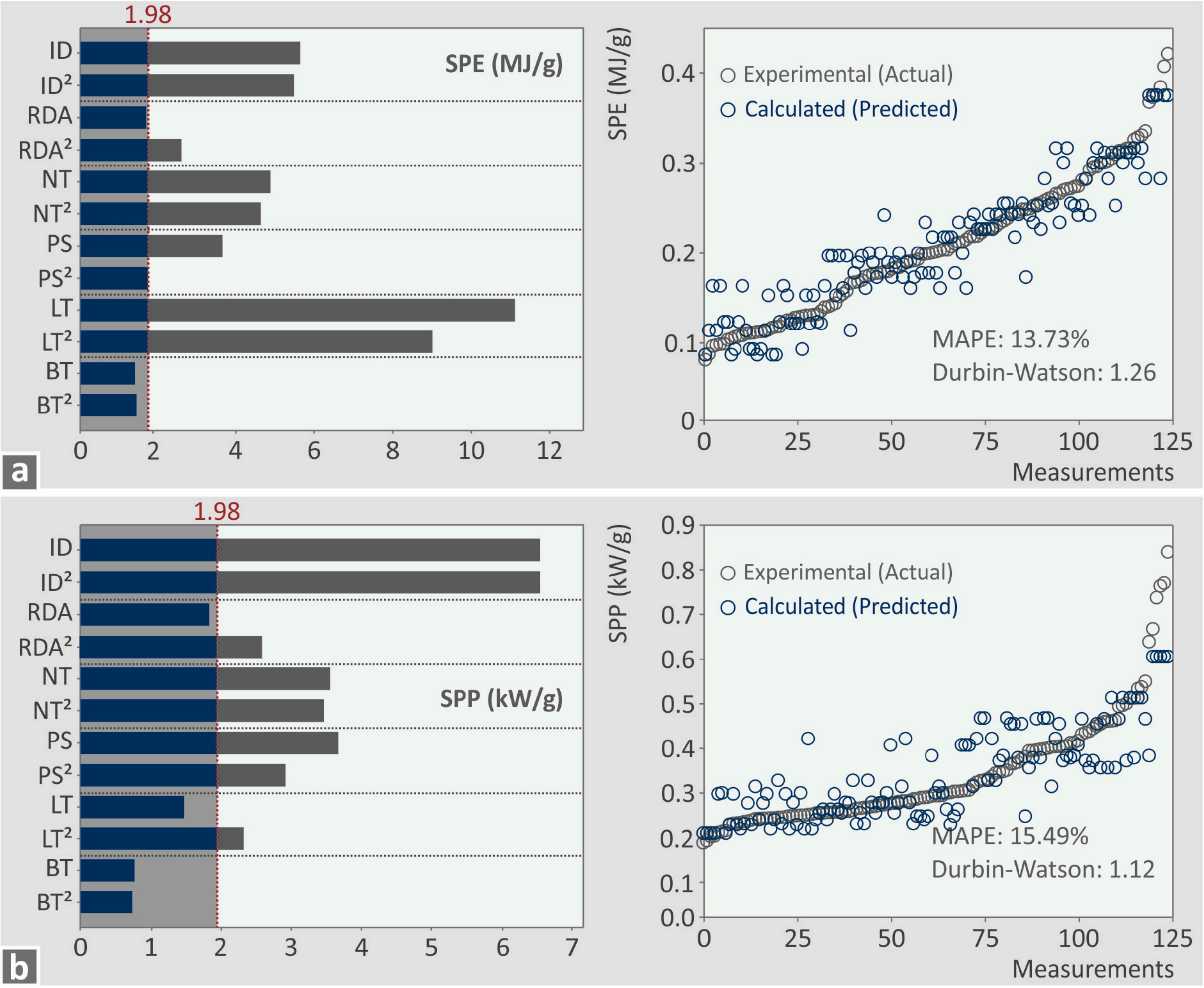

Figure S7. Pareto and experimental vs calculated charts: (a) SPE (MJ/g), (b) SPP (kW/g)

3.5. Confirmation run

Table S7. Measured Weight, Tensile Strength, Tensile Modulus of Elasticity and Tensile Toughness for each experimental run and five replicas per run for the Confirmation Runs.

A/A	Run	Weight (g)	sB (MPa)	E (MPa)	Toughness (MJ/m³)
1		2.088	65.52	248.44	8.30
2		2.087	66.81	255.81	9.06
3	26	1.992	64.68	253.09	8.49
4		2.077	62.34	272.42	8.44
5		2.182	65.95	270.27	8.82
6		0.868	28.79	179.51	3.84
7		0.875	27.81	194.98	3.83
8	27	0.850	28.96	189.95	3.89
9		0.855	27.54	190.25	4.00
10		0.837	27.91	187.62	3.86
	Min:	0.837	27.54	179.51	3.83
	Max:	2.182	66.81	272.42	9.06
	Average:	1.471	46.63	224.23	6.25

Table S8. Measured Printing Time, EPC, SPE, SPP for each experimental run and five replicas per run for the Confirmation Runs.

A/A	Run	Printing Time (s)	EPC (MJ)	SPE (MJ/g)	SPP (kW/g)
1		1172	0.633	0.303	0.259
2		1230	0.589	0.282	0.229
3	26	1244	0.638	0.320	0.258
4		1278	0.564	0.271	0.212
5		1174	0.574	0.263	0.224
6		302	0.082	0.094	0.312
7		282	0.068	0.078	0.277
8	27	297	0.065	0.077	0.259
9		307	0.068	0.079	0.257
10		288	0.061	0.073	0.253
	Min:	282	0.061	0.073	0.212
	Max:	1278	0.638	0.320	0.312
	Average:	757.40	0.334	0.184	0.254
